# Toward snowpack runoff decision support

**DOI:** 10.1016/j.isci.2022.104240

**Published:** 2022-04-12

**Authors:** Anne Heggli, Benjamin Hatchett, Andrew Schwartz, Tim Bardsley, Emily Hand

**Affiliations:** 1 Division of Atmospheric Sciences, Desert Research Institute, 2215 Raggio Parkway, Reno 89512, NV, USA; 2 University of California, Berkeley, Central Sierra Snow Laboratory, Soda Springs, 95728 CA, USA; 3 National Weather Service, Reno, 1000 Dandini Road, Reno 89512, NV, USA; 4 Department of Computer Science, University of Nevada, Reno, 1664 North Virginia Street, Reno 89503, Nevada, USA

**Keywords:** earth sciences, environmental event, environmental management, environmental monitoring, water resources engineering

## Abstract

Rain-on-snow (ROS) events are commonly linked to large historic floods in the United States. Projected increases in the frequency and magnitude of ROS multiply existing uncertainties and risks in operational decision making. Here, we introduce a framework for quality-controlling hourly snow water content, snow depth, precipitation, and temperature data to guide the development of an empirically based snowpack runoff decision support framework at the Central Sierra Snow Laboratory for water years 2006–2019. This framework considers the potential for terrestrial water input from the snowpack through decision tree classification of rain-on-snow and warm day melt events to aid in pattern recognition of prominent weather and antecedent snowpack conditions capable of producing snowpack runoff. Our work demonstrates how (1) present weather and (2) antecedent snowpack risk can be “learned” from hourly data to support eventual development of basin-specific snowpack runoff decision support systems aimed at providing real-time guidance for water resource management.

## Highlights

• A framework for a snowpack runoff decision support is introduced using station data
• Soil moisture data are used to identify periods of terrestrial water input
• Semi-automated quality control procedures improved model accuracy by up to 25.7%
• Snowmelt is not a primary source of runoff during rain-on-snow events

## Graphical abstract

**Figure:**
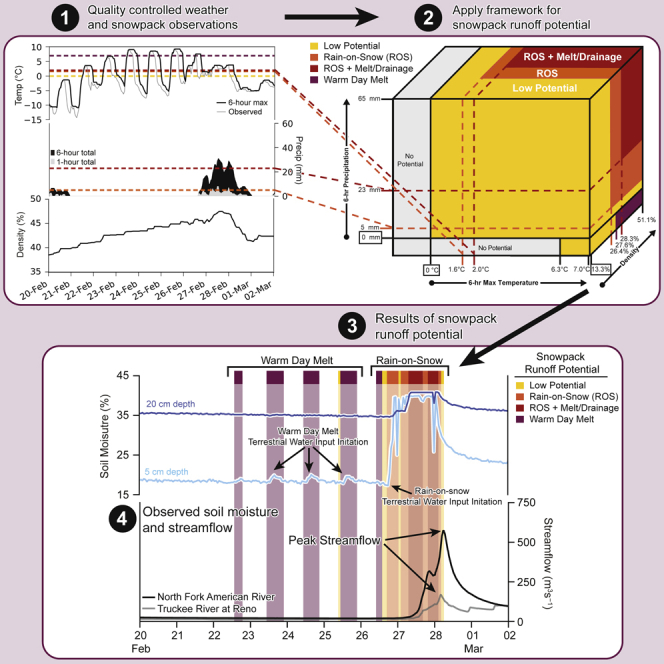

## Introduction

Reliable hydrometeorological data in mountain regions benefit society when applied to decision support tools at relevant scales, helping decision makers allocate valuable and limited resources to better prepare for potential flooding in order to protect life and property (Uccellini and Ten Hoeve, 2019; White et al., 2013; Ralph et al., 2014; Siirila-Woodburn et al., 2021). Agencies such as the National Weather Service (Uccellini and Ten Hoeve, 2019; NWS, 2020), National Oceanic and Atmospheric Administration (NOAA, 2020), and World Meteorological Organization (WMO, 2021) are transitioning from deterministic forecasts toward probabilistic forecasts with risk thresholds that communicate uncertainty to enable targeted messaging for their partners through impact-based decision support services. The National Weather Service provides forecasts, briefings, and watches, warnings and advisories to inform decision makers and the public of potential weather- and water-related hazards. Essential elements of information and impacts for probabilistic hazard information decision support services consider specific hazards, timing, peak conditions, as well as compounding and/or cascading impacts. Currently, there is no decision support service to provide situational awareness regarding the timing and peak conditions of changes in the snowpack during warm, windy, and wet winter storms. To address the lack of operational guidance on whether mountain snowpack will reduce runoff, act to enhance it, or have no effect (Brandt et al., 2022a), we propose the development of a snowpack runoff decision support framework, which aims to add another link in the chain of essential information for agencies like the National Weather Service to examine the likelihood and impact of midwinter rain-on-snow (ROS) runoff.

The snow-dominated Sierra Nevada is a major water source for California and western Nevada (He et al., 2016; Sterle et al., 2019). Forming part of the western margin of the North American Cordillera, the Sierra Nevada trends north-northwest to south-southeast and is approximately 640 km long and 110 km wide. It receives about half of its annual 1,580 mm precipitation in the core Northern Hemisphere winter months (December–February) (Chang et al., 2015) with over 70% falling as snow in the upper elevations (Lynn et al., 2020). Spring snowmelt from snow accumulated during winter provides approximately one third of California’s water supply (He et al., 2016) and is responsible for refilling reservoirs for domestic water supply (Dettinger and Anderson, 2015), hydropower generation (Vicuna et al., 2008), irrigation (Godsey et al., 2014), groundwater recharge (Jasechko et al., 2014), and recreation (Ligare et al., 2011).

The Sierra Nevada, like other maritime mountain ranges worldwide, is prone to ROS. ROS is an efficient generator of runoff that can produce 50%–80% higher peak flows than spring snowmelt (Kattlemann, 1997; Kattelmann and Dozier, 1999; Hatchett and McEvoy, 2018). As a consequence, rainfall and snowmelt together can produce greater floods than either rainfall or snowmelt alone (Harr, 1981; Kattlemann, 1997; Singh et al., 1997; Marks et al., 1998). However, skillfully forecasting ROS events remains a significant challenge (Musselman et al., 2018; Henn et al., 2020). The lack of operational guidance creates a need for tools to provide situational awareness and decision support in both transitional (ephemeral) and snow-dominated regions (Siirila-Woodburn et al., 2021; Hatchett et al., 2020; Uccellini and Ten Hoeve, 2019).

This long-standing historic decision support need is accelerating with recent research suggesting ROS-prone regions, including the Sierra Nevada, are approaching a period of “peak ROS”. Peak ROS results from the juxtaposition of a warming climate experiencing more precipitation falling as rain but before warming induces a persistent decline in snowpack volumes (Siirila-Woodburn et al., 2021). More frequent rainfall is projected to increase the magnitude and frequency of ROS events during the 21st century resulting in 20%– > 100% increase in runoff with the greatest ROS flood risk impacting the Sierra Nevada (Musselman et al., 2018). The second signal is a continuation of a historical trend in declining snow volumes with projected losses of 30%–85% by 2100 (Siirila-Woodburn et al., 2021). These warming-induced changes are compounded by natural interannual snowpack variability (Dettinger and Cayan, 1995; Cayan and Georgakakos, 1995). Despite interannual changes, there is a trend across the western US of increased midwinter snow melt (before peak SWE), but the role of ROS in midwinter snowmelt has not yet been analyzed (Musselman et al., 2021). The numerous challenges facing water resource managers as the region approaches “peak ROS” will be further exacerbated by an increasing demand for consumptive water uses (OECD, 2012), emphasizing the timely need for midwinter snowpack runoff decision support to optimize water resource management.

**Figure 1:**
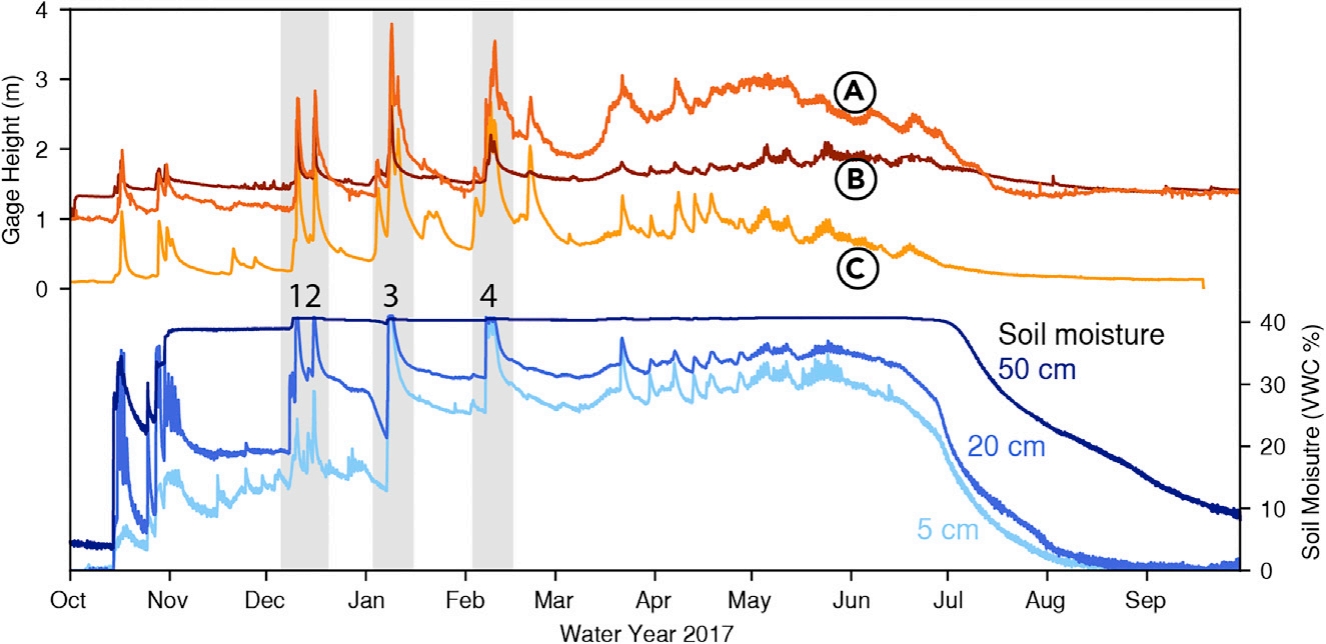
Correspondence between soil moisture change and streamflow response in nearby watersheds during four ROS events (1–4). Hourly soil moisture data (VWC %) at the Central Sierra Snow Laboratory for water year (WY) 2017 at 5 cm (light blue), 20 cm (medium blue), and 50 cm (dark blue) corresponds with stream flow response at three US Geological Survey gages: (A) Truckee River at Reno, (B) Ward Creek, and (C) North Fork of the American River at North Fork Dam.

**Figure 2:**
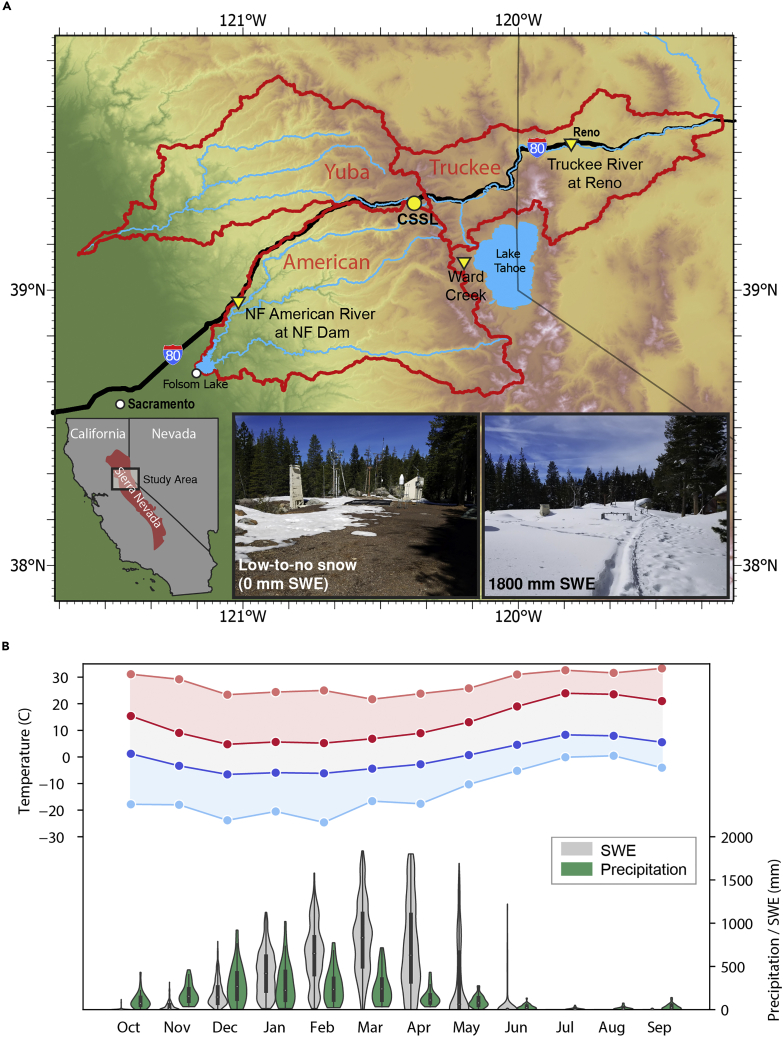
Study area location and climatological characteristics (A) Map of the Central Sierra Snow Lab. (B) Climograph based on water years 1988 through 2019 showing average (dark) and all-time (light) maximum (red) and minimum temperature (blue) on the left y axis. The right y axis shows the distribution of monthly accumulated precipitation (green) and snow water equivalent (grey).

Effective flood forecasting benefits from accurate meteorological predictions but potential hazard also depends on antecedent basin conditions (Oakley et al., 2017; Ralph et al., 2013; Georgakakos, 2006; Norbiato et al., 2008). Observational networks are key components in providing antecedent basin information for extreme event analysis to improve the understanding of physical processes linking hydrometeorological forecasts to impacts as well as real-time information for decision support (Hatchett et al., 2020; Sumargo et al., 2020; Ralph et al., 2013; Sterle et al., 2019; White et al., 2019). High temporal frequency resolution data from these networks are particularly valuable in providing event-based information. For example, hourly data from the US Department of Agriculture Natural Resource Conservation Service (NRCS) SNOw TELemetry (SNOTEL) network provides critical event-based information such as terrestrial water input (TWI) from the snowpack (Sutcliffe, 2014; Julander and Holcombe, 2005; Flint et al., 2008), snowmelt (Jennings and Jones, 2015), and density changes (Avanzi et al., 2014). At the University of California, Berkeley’s Central Sierra Snow Laboratory (CSSL) SNOTEL station, midwinter TWI from the snowpack corresponds not only with landfalling atmospheric rivers but also with increasing streamflow in nearby basins (Figure 2) on both the windward and the leeward sides of the Sierra Nevada (Figure 1; (Sterle et al., 2019; Hatchett et al., 2016)). The visible correlation between soil moisture and streamflow response is the motivation for our primary research question: can we use hourly data from existing snow monitoring networks to develop a decision support tool to aid in high-impact ROS events?

A knowledge gap in snow hydrology stems from a limited understanding of runoff timing and generation, specifically during ROS events (Colbeck, 1972; Kattelmann, 1985; Schneebeli, 1995; Kattelmann and Dozier, 1999; McCabe et al., 2007; Mazurkiewicz et al., 2008; Rössler et al., 2014). Midwinter ROS causes rapid grain growth due to capillary action concentrating the flow of water at the flow finger, aiding in the formation of preferential flow paths ahead of the leading edge of the newly wetted snow (wetting front) (Church, 1948; Colbeck, 1976; Marsh and Woo, 1984; Kattelmann, 1985; Marsh, 1987, 1999; McGurk et al., 1988; Kattelmann and Dozier, 1999; Hirashima et al., 2010; Katsushima et al., 2013). Once formed, the size and spatial extent of preferential flow paths varies depending on antecedent snowpack conditions and prior wetting events, which makes ROS runoff generation difficult to model (Marsh and Woo, 1984; Wever et al., 2014b). Current snowmelt models use a degree day algorithm (e.g. SNOW-17 (Anderson, 2006)), temperature index (e.g. HEC-HMS (Bartles et al., 2021), or more complex mass and energy balance equations (e.g. ISNOBAL (Marks et al., 2001); SNOWPACK (Wever et al., 2014b, a); SNODAS (Cho and Jacobs, 2020)) to calculate snowmelt. These models assume a uniform wetting front, do not account for preferential flow, and require snowmelt to occur to calculate TWI as a product of snowmelt. Cold content is a key parameter used in energy balance models to calculate the energy required to raise the temperature of the snowpack to 0°C and transition to latent heat exchange in order to melt snow. However, the snowpack does not need to become isothermal (0°C throughout) to transmit water during ROS (McGurk et al., 1988; Marsh and Woo, 1984). The dependency on these models to satisfy cold-content requirements could, in part, explain why snowmelt models struggle to reliably estimate event-based TWI during ROS (McCabe et al., 2007; Rössler et al., 2014; Clark et al., 2017; Hirashima et al., 2010).

To address the knowledge gap associated with during ROS events and support the generation of an empirically based snowpack runoff decision support framework, three fundamental methods were applied consecutively to build the snowpack runoff decision support framework (see STAR Methods for full details). First, we developed a TWI identification algorithm, which uses SNOTEL soil moisture data to classify periods of midwinter TWI (STAR Methods Terrestrial water input (TWI) identification algorithm and Filtering Data for Midwinter Snow-cover). Second, we developed Quality Assurance (QA) and Quality Control (QC) methods (STAR Methods Quality control (QC) and quality assurance (QA) methods and Quality control (QC) methods by observation type) to prepare hourly data for event-based learning as a key component of our exploration. Hourly SNOTEL data are not subject to the same quality control procedures as daily data and a skillful tool requires quality input data. The third method used decision tree classification (STAR Methods Quantification and statistical analysis) to simultaneously test the feasibility of automated classification of TWI drivers as ROS or warm day melt and measure the value of the QA/QC process by testing clean and raw data. Because we accurately classified TWI drivers, we then performed a frequency analysis of present weather and antecedent snowpack conditions for each TWI driver. This process aimed to demonstrate what can be learned about midwinter runoff generation from hourly data and develop the initial framework for a more broadly applicable snowpack runoff decision support tool.

Using data from CSSL spanning water years 2006–2019 (Figure 2), our paper aims to demonstrate how hourly data aid understanding of event-based changes and help to improve decision support through (1) the dissemination of runoff-relevant changes in the snowpack in real time, (2) pattern recognition of present weather and antecedent snowpack conditions that contribute to midwinter TWI, and (3) the provision of higher confidence validation data to advance the development of operational snowpack or hydrologic models. We demonstrate the feasibility of snowpack runoff decision support by developing methods for QA/QC, pattern recognition, and threshold identification at a single station as a testable framework for regional development. We anticipate this framework could be applied beyond the ROS problem and adapted for other environmental monitoring networks to aid the development of new or improved decision support for other natural hazards.

## Results

Our results are the outcome of cascading methods outlined in detail in the STAR Methods. The TWI identification algorithm (STAR Methods Terrestrial water input (TWI) identification algorithm), QA/QC of hourly SNOTEL data (STAR Methods Quality control (QC) and quality assurance (QA) methods and Quality control (QC) methods by observation type), and manual identification of TWI drivers as ROS or warm day melt (STAR Methods Target variable: ROS or warm day melt TWI) were used in conjunction to test the feasibility of automated decision tree classification of TWI driver and measure the value of data QA/QC (STAR Methods Quantification and statistical analysis). The decision tree classification model proved accurate and a frequency analysis was performed for over the midwinter training data (WYs, 2008–2019; STAR Methods Filtering data for midwinter snow-cover) to aid in pattern recognition of present weather and snowpack conditions for ROS and warm day melt TWI. Access to clean hourly data and the results from these methods demonstrate what can be learned to minimize the ROS runoff knowledge gap. Finally, the results from the frequency analysis were translated into the preliminary conceptual snowpack runoff decision support framework. The successive results are described in detail in subsequent sections.

### Soil moisture data can be applied to identify terrestrial water input (TWI)

The TWI identification algorithm developed with SNOTEL soil moisture data to identify periods of TWI (STAR Methods Terrestrial water input (TWI) identification algorithm) resulted in a total of 782 h of TWI with maximum soil moisture increases of up to 21% in 1 h (Feb 13, 2019). During one exceptional ROS event, TWI occurred for up to 48 consecutive hours and soil was saturated at all three depths for 24 consecutive hours (Jan 7–9, 2017). At least, 6 h of continuous TWI occurred on 25 days, all associated with ROS events. In contrast to ROS, warm day melt-driven TWI lasted less than 3 h on average but not more than 5 h.

Periods of TWI identified by the algorithm were manually classified as ROS or warm day melt (STAR Methods Target variable: ROS or warm day melt TWI). Of the 782 h of TWI, 499 h coincided with ROS, 264 h coincided with warm day melt, and 19 h could not be explained by ROS or warm day melt. Of the anomalous 19 h, 16 h were misidentified by the algorithm and the remaining three instances could not be explained by precipitation or temperature data. The 19 unidentifiable data points were excluded from the study to address principal drivers of TWI from ROS and warm day melt. From the remaining 763 data points, ROS TWI accounted for 65.4% of all TWI events and warm day melt accounted for the remaining 34.6%.

### Automated classification of TWI driver

#### Clean data improved model accuracy by up to 25.7% when classifying TWI drivers

The decision tree classification performed with raw and clean data quantified the added value the QA/QC method had in classifying TWI drivers (STAR Methods Quantification and statistical analysis). The cross-validation of the model tested on each set of two consecutive water years and trained on remaining years for clean and raw data (STAR Methods Decision Tree Classifier Criteria) demonstrates the value of the QA/QC methods (STAR Methods Quality control (QC) and quality assurance (QA) methods and Quality control (QC) methods by observation type) applied to the hourly data (Table 1). The model performance was measured with accuracy (number of correct classifications) and F1 score (harmonic mean of the precision and recall) (Pedregosa et al., 2011). On average, clean data were 95.5% accurate (standard deviation of 4.4%) with an average F1 score of 0.96 (standard deviation of 0.03). Raw data performed consistently worse with an average accuracy of 84.0% (standard deviation of 5.3%) and an average F1 score of 0.859 (standard deviation of 0.08). 2009 and 2010 performed the same because the data during TWI events did not need significant correction and had a very small dataset to test on. On the other hand, WY 2018 and 2019 required extensive quality control during periods of TWI that improved the model accuracy by 25.7%. On average for the study period, the clean data were 13.7% more accurate.

**Table 1:** Decision tree classification model cross-validation results

Test data parameters				Clean data		Raw data	
Water years	Data points	Data size	ROS data size	Accuracy	F1 score	Accuracy	F1 score
2006 & 2007	212	27.8%	62.3%	0.976	0.981	0.825	0.871
2007 & 2008	125	16.4%	36.8%	0.992	0.989	0.816	0.875
2008 & 2009	64	8.4%	40.6%	1.000	1.000	0.891	0.868
2009 & 2010	82	10.7%	40.2%	0.951	0.943	0.951	0.943
2010 & 2013	124	16.3%	26.6%	0.960	0.928	0.823	0.667
2013 & 2014	134	17.6%	45.5%	0.948	0.942	0.821	0.774
2014 & 2015	73	9.6%	80.8%	0.863	0.911	0.836	0.891
2015 & 2016	46	6.0%	91.3%	0.891	0.943	0.804	0.883
2016 & 2017	191	25.0%	88.5%	0.979	0.988	0.853	0.913
2017 & 2018	184	24.1%	90.2%	1.000	1.000	0.875	0.927
2018 & 2019	99	13.0%	80.8%	0.939	0.962	0.747	0.839

A model was built and tested on two consecutive water years; the total test data side in number of points and percent of data are detailed along with the percentage of TWI events that were classified as ROS. Accuracy (number of correct classifications) and F1 score (harmonic mean of the precision and recall) is provided as a measure of model improvement for clean versus raw data.

Our results demonstrate that randomized selection of test and training data for unbalanced data like is collected at CSSL will not necessarily build the best model. The size of the test data is not consistent between water years because the snow cover season is not consistent. Similarly, the results between water years are variable because the number of ROS or warm day melt events is not consistent. Criteria for the test and training data split benefits from a comprehensive understanding of the dataset (e.g. length of the snow covered season, number of TWI events, number or ROS events, or warm day melt events).

#### Model results

The decision tree classification trained on WYs 2008–2019 and tested against WYs 2006 (wet year; four events) and 2007 (dry year; two events) was selected to develop the framework model. The 2006 and 2007 test parameters had the highest number of test data points and the most representative distribution of ROS and warm day melt events compared to the entire dataset; 62.3% (132 data points) were associated with ROS and 37.7% (80 data points) were associated with warm day melt. The clean data model accurately classified 97.6% of TWI drivers with an F1 score of 0.981 while the raw data model was 82.5% accurate with F1 score of 0.871 (Table 1).

**Figure 3:**
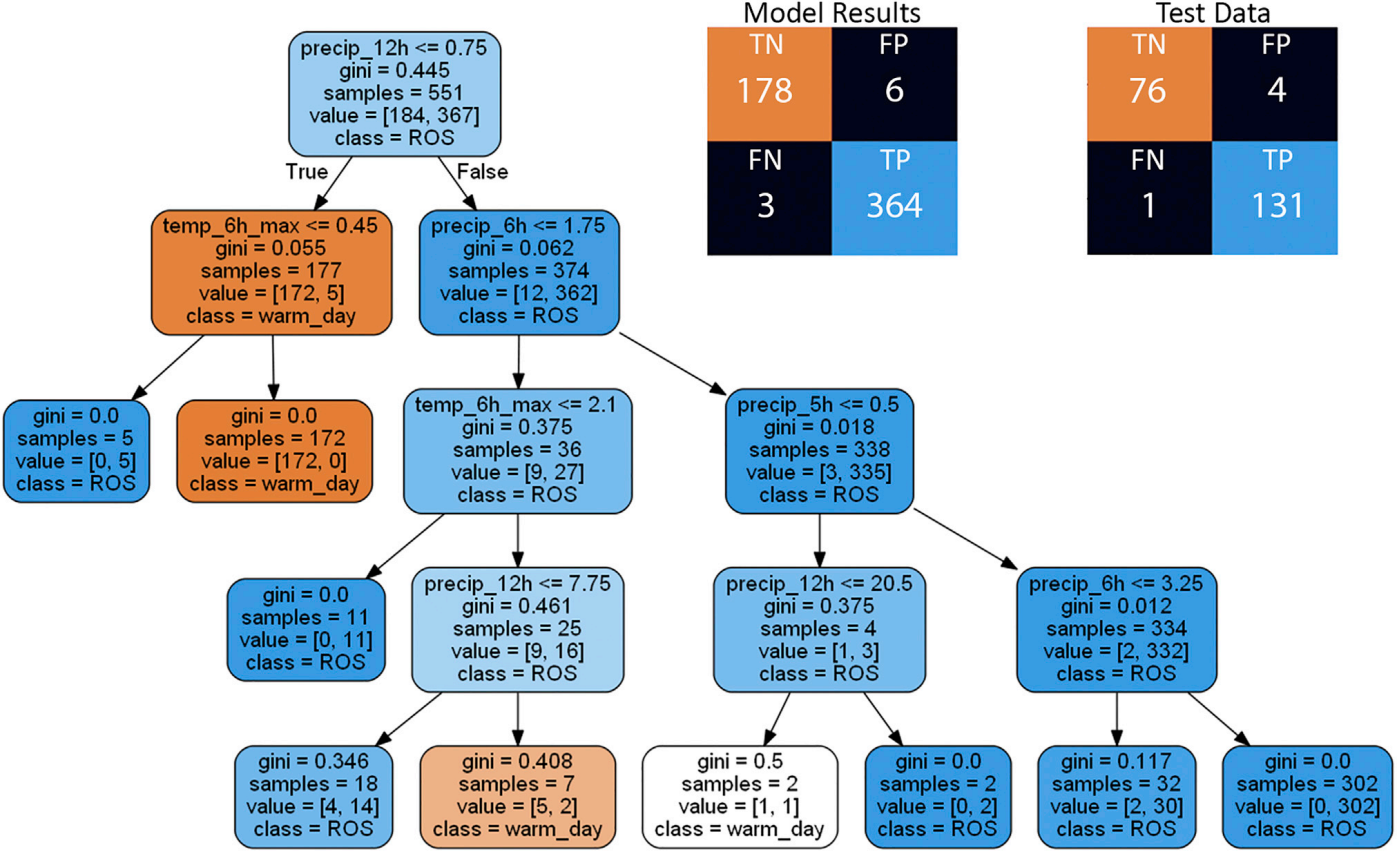
Decision tree classification of ROS and warm day melt events from the cleaned data Decision tree classification model (STAR MethodsDecision tree classification) for the cleaned data and confusion matrix for the results of the model and test data illustrating the number of true positives (TP), true negatives (TN), false positives (FP), and false negatives (FN) of the predicted values against the actual values with true identifying ROS and false identifying warm day melt TWI. See STAR MethodsDecision tree classification and Decision tree classifier criteria for further detail.

The classification model developed with clean data has four impure leaf nodes where a total of six warm day melt events and three ROS events were incorrectly classified (Figure 3). The confusion matrix shows the number of true positives (TP), true negatives (TN), false positives (FP), and false negatives (FN) of the predicted values against the actual values with true identifying ROS and false identifying warm day melt TWI for the model results and test data. The root node split identifies the variable that best splits the data and the model identified 12-h precipitation totals less than or equal to 0.75 mm for the root node. Following the false classifications down the right-most side of the tree, the decision tree correctly classified 335 out of 338 samples as ROS by looking for and 6-h precipitation totals over 1.75 mm. The first internal node to the left of the root node correctly identified all 172 samples as warm day melt when 6-h maximum temperatures greater than 0.45°C. The five samples classified as ROS when 12-h precipitation was less than 0.75 and temperatures less than 0.45°C, the samples were manually identified as a lagged ROS release from the February 13–14, 2019 ROS event.

#### Raw data are less reliable

**Figure 4:**
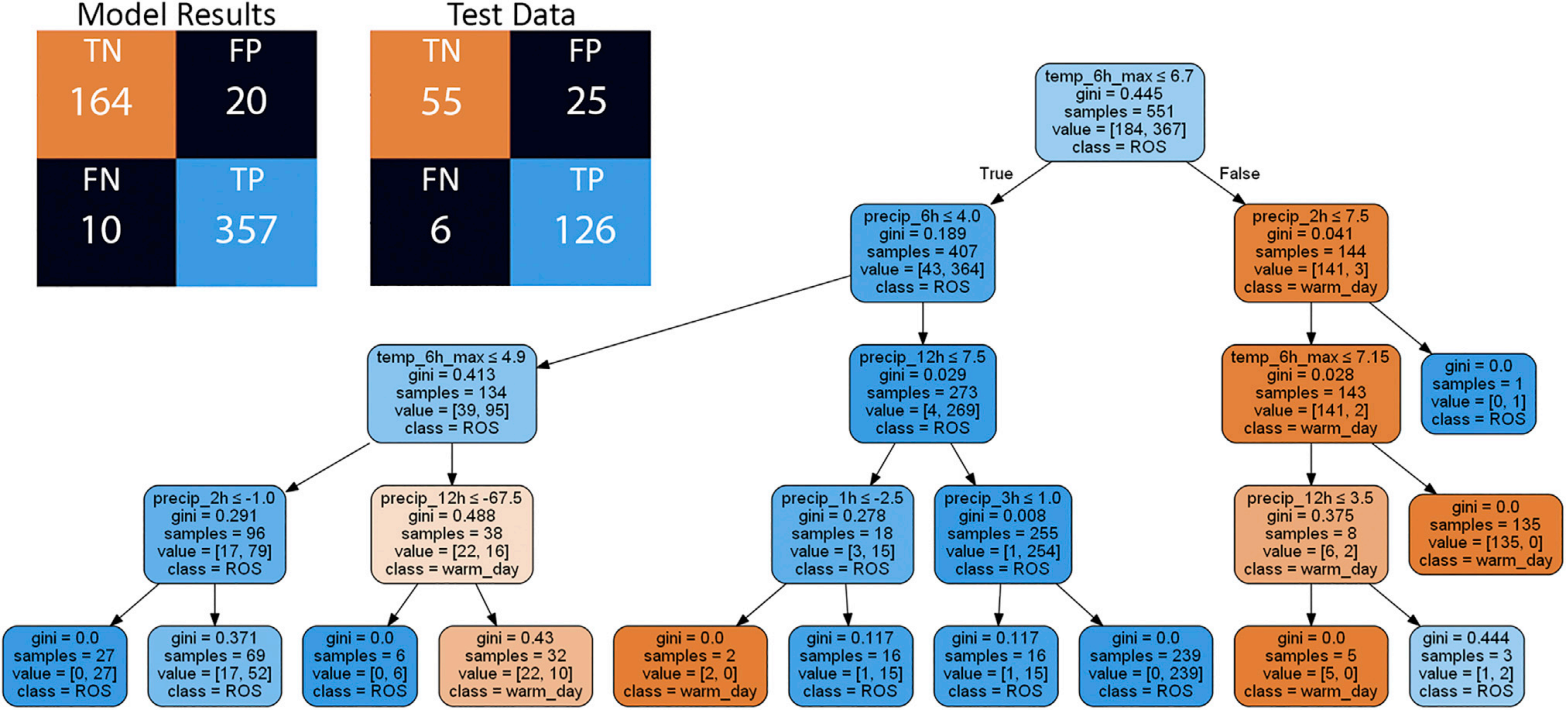
Decision tree classification of ROS and warm day melt events from the raw data Decision tree classification model (STAR MethodsDecision tree classification) for the raw data and confusion matrix for the results of the model and test data illustrating the number to true positives (TP), true negatives (TN), false positives (FP), and false negatives (FN) of the predicted values against the actual values with true identifying ROS and false identifying warm day melt TWI. See STAR MethodsDecision tree classification and Decision tree classifier criteria for further detail.

The decision tree classification model was also built with raw data under the same criteria. The raw decision tree model has five impure nodes where 20 warm day melt samples and 10 ROS samples were incorrectly classified (Figure 4). There are three examples of data issues impacting the model. Three leaves with negative precipitation values demonstrate the impact of diurnal flutter causing false decreases in precipitation. The classification of 12-h precipitation less than −67.5 mm was the result of the January 7–9, 2017 ROS event that flooded the station and damaged the pressure transducer. Events with 6-h maximum temperatures greater than 6.5°C and more than 7.5 mm of precipitation in the last 2 h were classified as ROS, but this is an example of warm day melt that caused a snow plug release (see STAR Methods 3.4.2 for information about snow plug releases).

### Present weather and antecedent snowpack

#### Frequency analysis

The automated classification of TWI drivers as ROS or warm day melt (STAR Methods Decision Tree Classifier Criteria) derived from the TWI identification algorithm (STAR Methods Terrestrial water input (TWI) identification algorithm), QA/QC of hourly SNOTEL data (STAR Methods Quality control (QC) and quality assurance (QA) methods and Quality control (QC) methods by observation type), and manual identification of TWI drivers as ROS or warm day melt (STAR Methods Target Variable: ROS or Warm Day Melt TWI) proved both feasible and reliable. Identifying TWI and classifying the driver as ROS or warm day melt facilitates pattern recognition of present weather and antecedent snowpack conditions with the potential to generate runoff for each TWI driver. To build the snowpack runoff decision support framework, the frequency of present weather and antecedent snowpack conditions were examined for the training data (WYs 2008–2019) using the manually classified TWI driver. The 6-h maximum temperature and 6-h precipitation totals provide the distribution and frequency of present weather conditions while snowpack density as a percent (NRCS, 2014) shows the distribution and frequency of antecedent snowpack conditions during periods of TWI. It is worth noting that warm day melt is correlated with incoming solar radiation, which is the primary driver of snowmelt; however, as solar radiation is not commonly measured at SNOTEL stations, temperature is used as a proxy (Painter et al., 2012). The ROS classification was subset to include ROS + melt/drainage defined as all ROS events with at least a 2 mm loss of SWE in the last one hour. Of the 454 hours of ROS TWI, only 45 hours were coupled with SWE loss, accounting for 9.9% of ROS TWI and 5.8% of all TWI identified in this study. These results provide the first indication that snowmelt is not a primary source of midwinter runoff.

**Figure 5:**
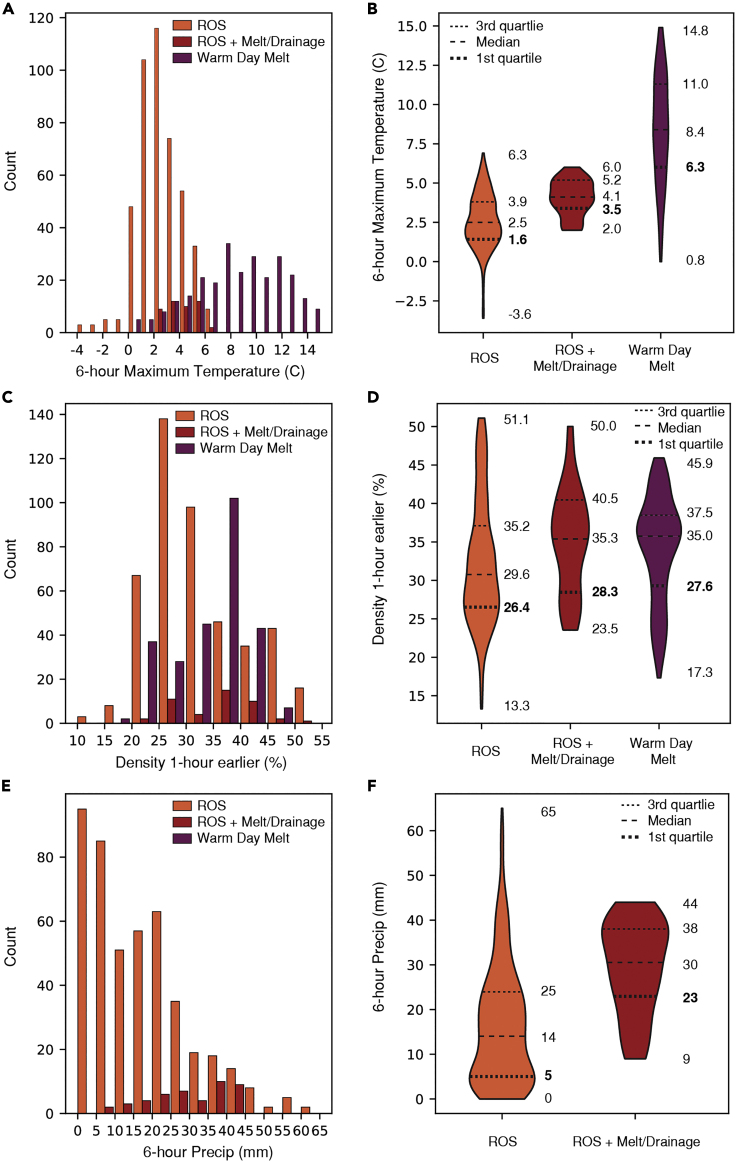
Distributions of present weather and snowpack conditions for rain-on-snow (ROS), rain-on-snow plus snow water equivalent loss (ROS + melt/drainage), and warm day melt (A) Histogram showing the distribution of 6-h maximum temperature (°C) for ROS (orange), ROS + melt/drainage (red), and warm day melt (purple) events. (B) Box-percentile plots showing the distribution of ROS, ROS + melt/drainage, and warm day melt events for varying temperatures. Dashed lines represent first quartile, median, and third quartile values with first quartile in bold to draw the connection to the development of the framework in Snowpack runoff decision support framework section. (C and D) As in panels (A) and (B), but for density (%). (E) and (F) As in panels (A) and (B), but for 6-h precipitation (mm).

ROS-driven TWI events during the training data period had 6-h maximum temperatures ranging from −3.6°C to 6.3°C with an interquartile range of 1.6°C–3.89°C (Figures 5A and 5B). The maximum 6-h temperature for ROS + melt/drainage ranged from 2.0°C to 6.0°C with interquartile values of 3.4°C and 5.2°C. Warm day melt had 6-h maximum temperatures range of 0.8°C–14.8°C and interquartile range of 6.3°C–11.0°C.

ROS events produced TWI when snowpack densities were as low as 13.3% and up to 51.1% with an interquartile range of 26.4%–35.2% (Figure 5C and D). Snowpack density was also higher for ROS + melt/drainage events, ranging from 23.5% to 50.0% with an interquartile range of 28.3%–40.5%. Density values 1-h prior to warm day melt-driven TWI ranged from 17.3% to 45.9% with an interquartile range of 27.6%–37.5%.

ROS TWI only occurred with measurable precipitation when temperatures were greater than 0°C (Figure 5E). 6-h precipitation totals during ROS TWI ranged from 0 to 65 mm with an interquartile range of 5–24 mm (Figure 5F). When 6-h precipitation totals were 0 mm, TWI observations were associated with lagged ROS TWI (February 14, 2019). During ROS + melt/drainage, 6-h precipitation totals ranged from 9 to 44 mm with an interquartile range of 23–38 mm.

#### Snowmelt is not a primary source of runoff in deeper snowpacks

**Figure 6:**
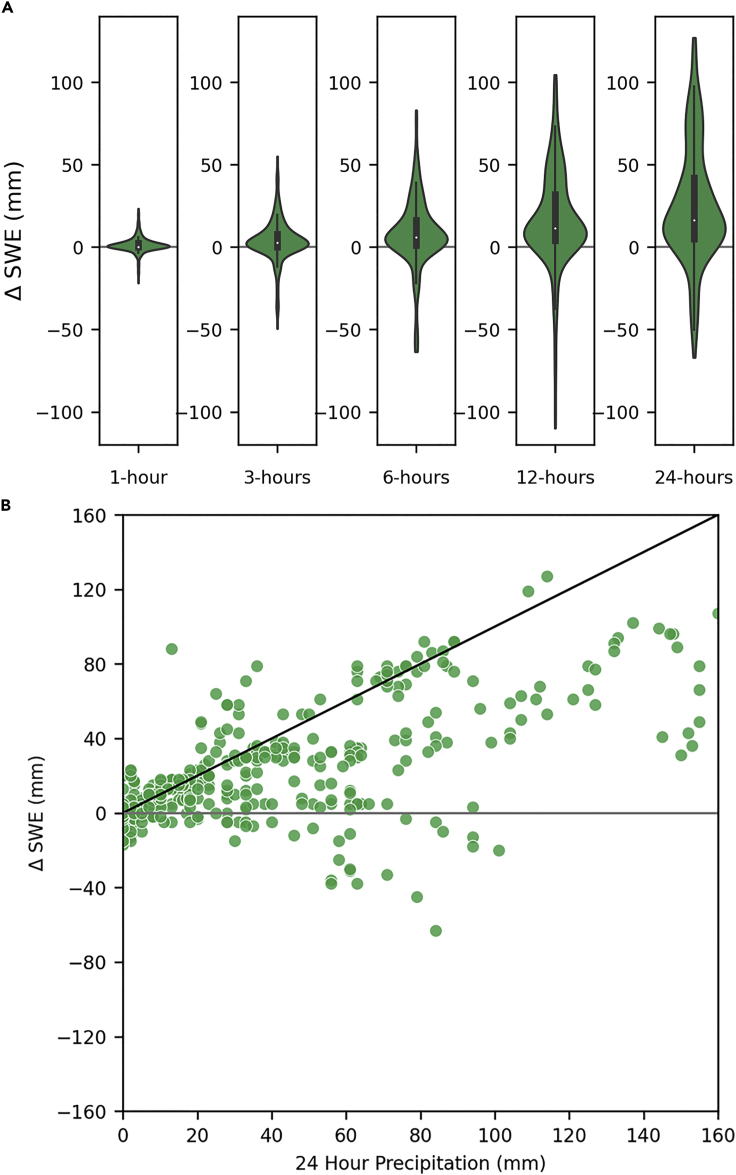
Change in SWE from one to 24 h during ROS (A) 1-h, 3-h, 6-h, 12-h, and 24-h total change in SWE (mm) during ROS events. (B) 24-h change in SWE (mm) versus 24-h precipitation totals (mm) with the black 1:1 line indicating periods when the snowpack accumulated all of the precipitation and a gray line when SWE was lost over 24 h.

Our findings are consistent with previous findings that snowmelt is not a primary source of runoff during ROS events in deeper snowpacks (Mazurkiewicz et al., 2008; Singh et al., 1997; Whitaker and Sugiyama, 2005). At least, 75% of 1-h, 3-h, 6-h, 12-h, and 24-h SWE changes resulted in an increase in SWE during ROS TWI (Figure 6A). Therefore, the snowpack can accumulate SWE while simultaneously producing runoff. Notably, only 16% (80 of 499 h) of ROS TWI occurred with 24-h SWE loss. 38.7% (31 of 80 h) of 24-h SWE loss occurred during the January 2017 and February 2017 ROS events (Figure 6B). These events require additional analysis to differentiate the draining of liquid water following snowpack charging—where water is transiently stored in the snow matrix (Marsh and Woo, 1984; Brandt et al., 2022b)—from actual snowmelt.

#### Daily rainfall thresholds that produce TWI

**Figure 7:**
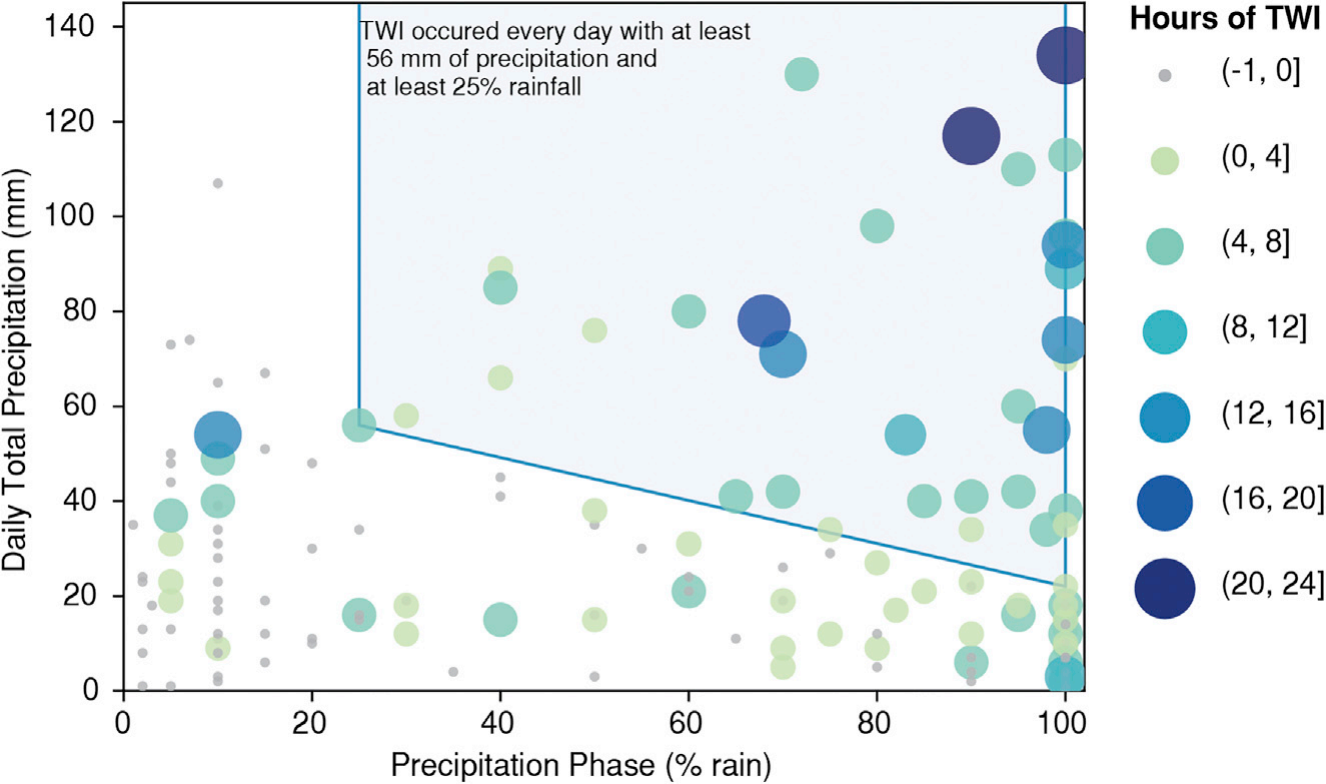
Duration of TWI as a function of total daily precipitation and percent rain Daily total precipitation and precipitation phase as percent (%) rain from manual observations from the Central Sierra Snow Laboratory and SNOTEL-derived cumulative hours of TWI per day and total precipitation.

There is a general relationship between precipitation phase and intensity with TWI. The CSSL manual observations include daily precipitation phase values as percent rain and percent snow. The percent rain values were used to analyze hours of TWI as a function of daily total rainfall (precipitation and percent rain; Figure 7). TWI occurred if at least 22 mm of precipitation fell as rain. This relationship was not observed when less than 25% of precipitation falls as rain. However, 24-h precipitation totals of at least 56 mm with at least 25% rain produced a TWI signal in the soil moisture response. As storms include a greater fraction of precipitation falling as rain, less total precipitation was required to produce TWI. Days with 50% rain required at least 38 mm to fall as rain whereas on days with 100% rain only 22 mm of precipitation was necessary.

### Snowpack runoff decision support framework

#### Preliminary snowpack runoff decision support framework

**Figure 8:**
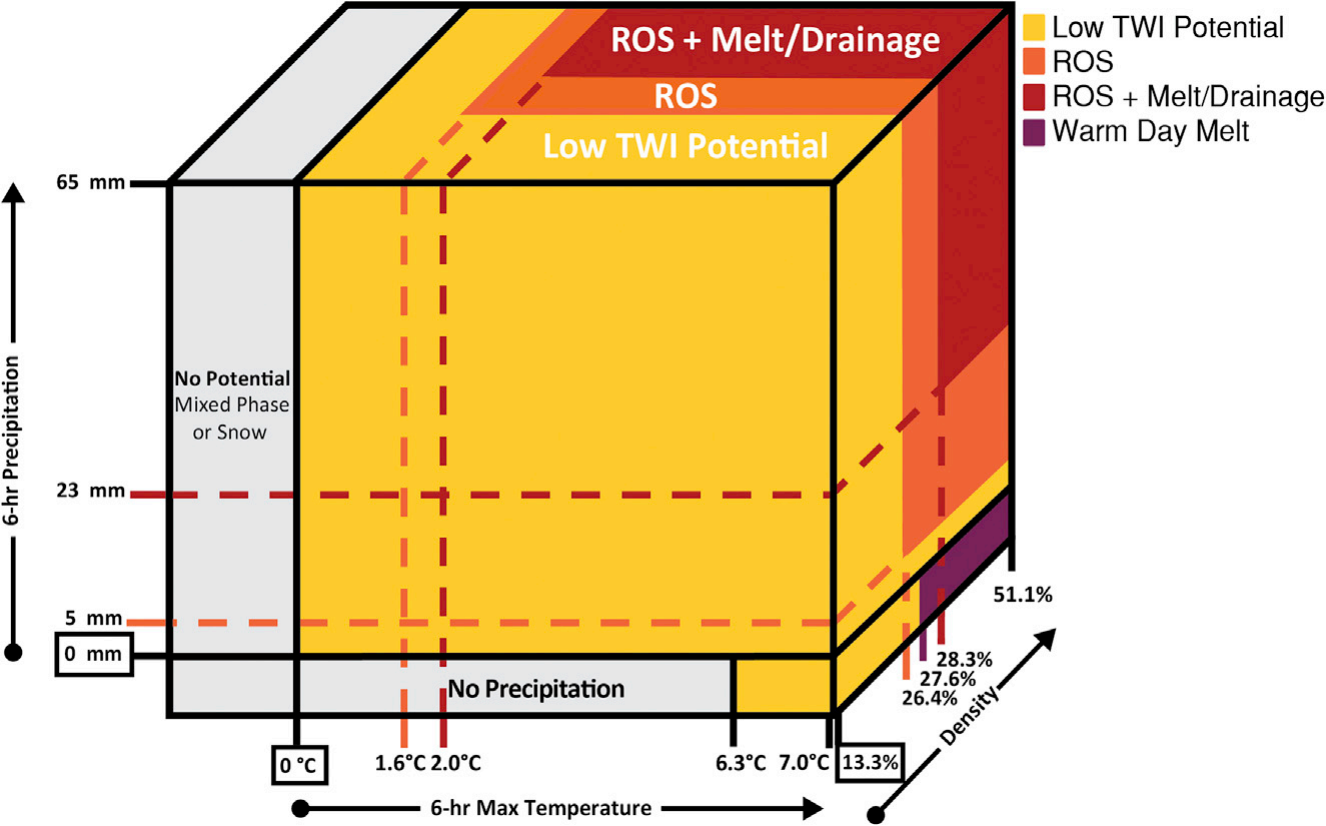
Conceptual snowpack runoff decision support framework Snowpack runoff decision support conceptual framework developed through the application of first-quartile 6-h precipitation, 6-h maximum temperature, and density 1-h earlier as indicators of low, ROS, ROS + melt/drainage, or warm day melt TWI potential.

We present a first-step, conceptual snowpack runoff decision support framework guided by the knowledge acquired through the development of a TWI identification algorithm, high confidence hourly data via QA/QC procedures, decision tree classification of ROS and warm day melt-driven TWI, and frequency analysis of present weather and antecedent snowpack conditions. TWI is the first indication of snowpack runoff. Identifying and classifying periods of TWI lays the foundation for snowpack runoff decision support. To build upon this, we selected first quartile values for 6-h maximum temperature (Figure 5B), 6-h precipitation (Figure 5F), and snow density 1-h prior to TWI (Figure 5D) as preliminary indicators of potential TWI. These indicators were integrated into the conceptual three-dimensional snowpack runoff decision support framework (Figure 8). “Low Potential” refers to values when TWI potential was below first quartile values. Warm day melt was defined by non-ROS TWI as a result of 6-h maximum temperatures of at least 6.3°C and density of at least 27.6%. The potential for ROS-induced TWI was established with as little at 5 mm of precipitation, maximum temperatures greater than 1.6°C, and density of at least 26.4%. The potential for TWI during ROS events increases when SWE loss can occur as a result of either snowmelt or drainage of transiently stored rainwater. The potential for ROS + melt/drainage was defined when 6-h precipitation totals exceeded 23 mm, 6-h maximum temperatures were greater than 2.0°C, and the snowpack density was at least 28.3%.

There was no evidence of midwinter precipitation occurring above 7.0°C during the period of study. A midwinter ROS event when temperatures are greater than 7.0°C would be unprecedented in recent history as the 1997 New Year’s event had daily maximum temperatures at or below 7.0°C (Osterhuber and Schwartz, 2021). Though it is plausible for threshold to be crossed some day since the CSSL does experience rainfall in the fall months above 7.0°C, identifying the current precipitation ceiling is an important metric. Crossing this threshold could produce greater runoff as the rainfall would carry more energy to melt snow. This further emphasizes the value of reliable hourly data, which would make it possible to analyze larger scale changes like an increase in the midwinter precipitation temperature ceiling.

**Figure 9:**
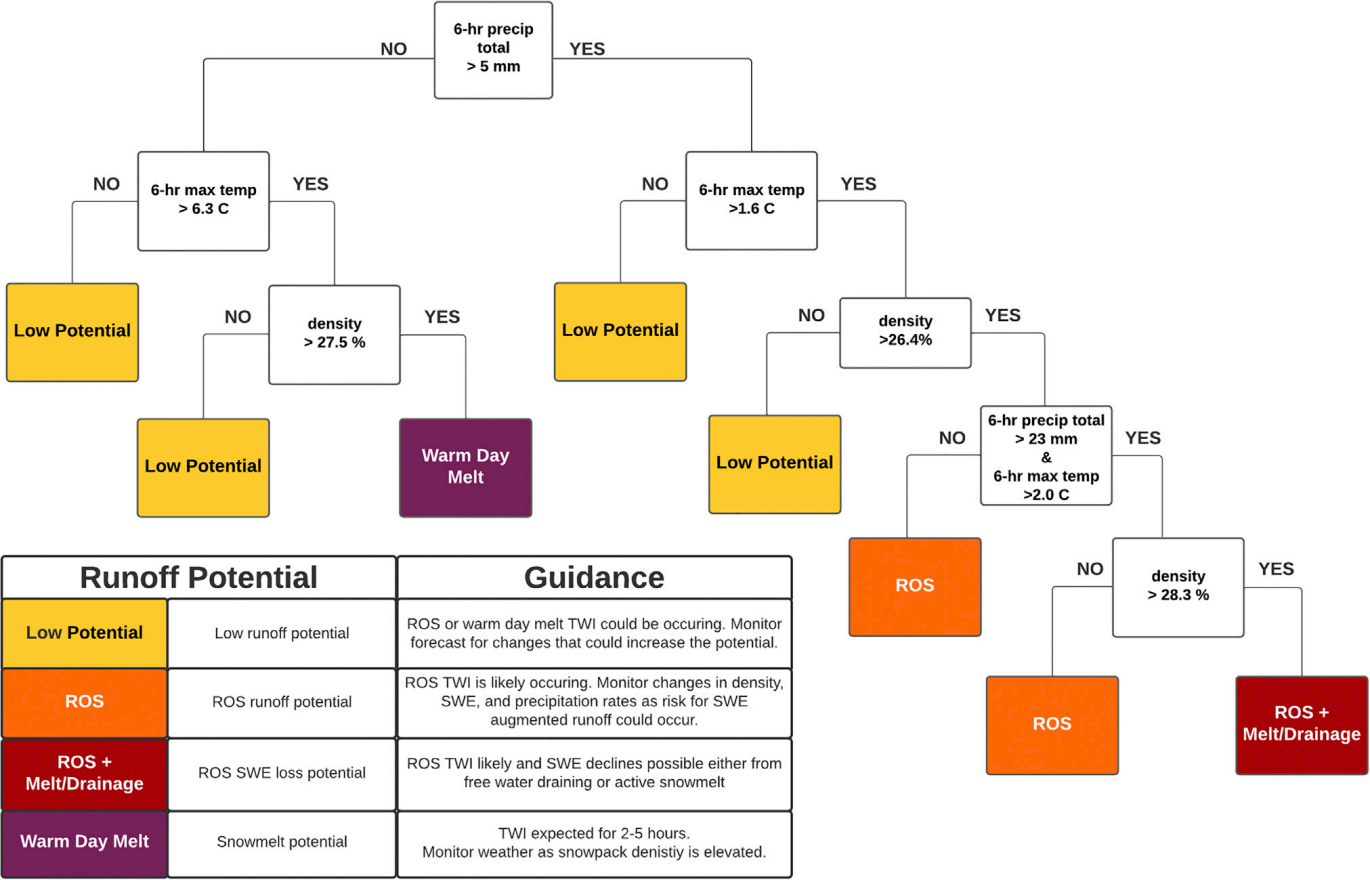
Decision tree visualization of the snowpack runoff decision support framework developed at the Central Sierra Snow Laboratory with color scale indicating TWI potential

We translated the conceptual snowpack runoff decision support framework into a decision tree with an index to simplify the identification of snowpack runoff potential for decision makers (Figure 9). This initial step toward snowpack runoff decision support demonstrates how hourly data can be fed into a system to improve multidimensional situational awareness. Impactful decision support tools like the Air Quality Index (Agency, 1999) include a quantitative color scale but only provide one-dimensional information (particulate matter). Multidimensional advisories like the National Weather Service Heat Index (Hawkins et al., 2017) and Avalanche Danger Scale (Statham et al., 2010) provide a cohesive measure of danger through qualitative color scales. We applied qualitative and quantitative communication methods by including a color scale for the TWI potential and values for each TWI indicator.

#### Example application of preliminary framework

**Figure 10:**
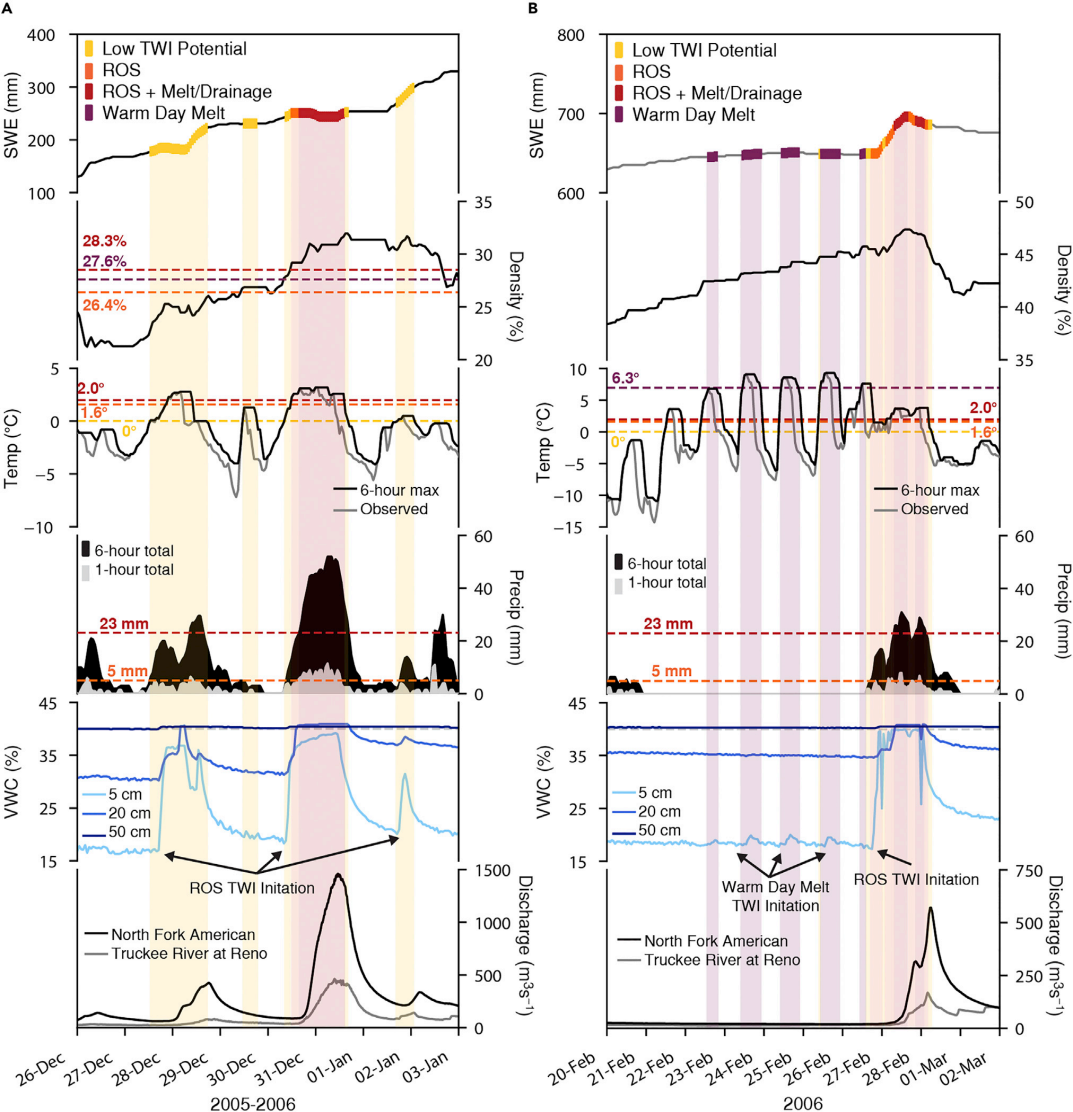
Example applications of the preliminary snowpack runoff decision support framework The decision tree TWI potential thresholds applied to (a) December 26, 2005 through January 3, 2006 and (b) February 20–March 2, 2006. The first subpanel of each plot shows SWE (mm) colored by TWI potential as low potential (yellow), ROS (orange), ROS + melt/drainage (red), and warm day melt (purple). The second subpanel shows the snowpack density (%) with corresponding TWI potential thresholds with representative colors. The third subpanel shows observed (gray) and 6-h maximum (black) air temperatures (°C) with corresponding thresholds and the fourth panel shows 1-h (filled gray) and 6-h (filled black) precipitations totals (mm) with the 6-h precipitation corresponding TWI potential thresholds. The fifth subpanel shows volumetric water content (%) from the soil moisture sensors at 5 cm (light blue), 20 cm (medium blue), and 50 cm (dark blue) depths. The sixth subpanel shows streamflow (m^3^s^−1^) at two US Geological Survey gages: North Fork of the American River at North Fork Dam (black) and Truckee River at Reno (gray).

As an example of the application of the preliminary snowpack runoff decision support framework, we applied the decision tree thresholds to two periods with three ROS events from WY2006 of the test dataset (Figure 10). From December 26, 2005 through January 3, 2006, two ROS events both produced TWI resulting in increases in soil moisture (Figure 10A). However, the ROS TWI potential was low because snowpack density values were below 26.4% and therefore did not qualify as ROS potential. ROS TWI when the snowpack density is low could be an indication of the formation or preferential flow paths as a uniform wetting front would increase the liquid water content to capacity prior to TWI (Marsh and Woo, 1984; Colbeck, 1976). This highlights an area to improve the framework since density may not be as critical in dictating TWI potential as rainfall intensity and totals due to the formation of preferential flow paths. The 2006 New Year’s Day ROS event had widespread impacts in the American, Yuba, and Truckee watersheds (CNRFC, 2021). The snowpack runoff decision support framework correctly indicated the potential to produce ROS TWI and later ROS + melt/drainage TWI. The second analysis period (February 20–March 2, 2006) had a ROS event proceeded by five days with no precipitation and maximum temperatures over 6.3°C with the potential for warm day melt (Figure 10B). By the second day of warm day melt potential conditions being met, the 5 cm soil moisture sensor registers diurnal melt from the snowpack. Knowing that the snowpack is at a state of active melt ahead of a potentially warm storm would indicate that snowpack is ready to transmit water and potentially contribute to TWI. On February 27, 2006, a ROS event began at the CSSL and within two hours of precipitation initiating, the soil moisture sensors registered increases. TWI potential increases to ROS + melt/drainage and all three soil moisture sensors measure a period of saturation when the TWI input rate is greater than the soil infiltration rate. While this example demonstrates the utility of the approach to identify present weather and antecedent snowpack conditions that could produce TWI, it also highlights the thresholds that miss TWI and can be used as guidance for further calibration of the framework.

## Discussion

### Development of a cascading workflow: data QA/QC, TWI identification algorithm, and pattern recognition

The daily SNOTEL data product provides a valuable tool for advancing process-based knowledge of snow runoff generation and timing resulting in improved model accuracy and remote sensing products (Lundquist et al., 2015; Chen et al., 2019; Song et al., 2021). Readily available, high confidence, and comprehensive hourly data help streamline research efforts, allowing investigators to focus on results and analysis to close the ROS runoff knowledge gap (Rössler et al., 2014; McCabe et al., 2007; Brandt et al., 2022a). The semi-automated QA/QC approach represents a step forward in achieving this goal by reducing the time required to clean data by leveraging automated processes during more predictable periods, allowing the data reviewer to focus on events and flagged data anomalies. While we will continue to apply the QA/QC methods to the SNOTEL network, we recommend it also be applied to other observational networks both to further improve it and to identify additional weaknesses.

Our research found soil moisture data to be useful for identifying the timing of TWI (Cardell-Oliver et al., 2005; Julander and Holcombe, 2005; Flint et al., 2008; Bales et al., 2011; Sutcliffe, 2014). Because hourly soil moisture data have already been reviewed by NRCS staff, expanding the application of the TWI identification algorithm to other SNOTEL station could be implemented immediately. TWI identification could be beneficial for NWS hydrologists and decision makers by notifying them of active TWI in near-real time, especially when interpreted in tandem with data from other hydrometeorological networks (Hatchett et al., 2020). With the addition of quality-controlled hourly data for the remaining parameters from other stations (precipitation, snow depth, and snow water equivalent), applying the remaining methods could improve the pattern recognition of antecedent snowpack conditions and present weather that produce TWI regionally rather than at a single location.

Augmenting surface station-based data with information regarding present atmospheric conditions is an important component of early warning or real-time information systems by providing situational awareness (Hatchett et al., 2020) and contributing to impact-based decision support (Uccellini and Ten Hoeve, 2019) ahead of forecasting extreme events. To show our identified ROS-induced TWI events have common ingredients with ROS events, we created synoptic composites using daily averages from the National Center for Environmental Prediction’s 36 km horizontal resolution North American Regional Reanalysis (Mesinger et al., 2006). We selected 17 unique storm events (for multi-day events, the first day was used) with at least six hours of continuous TWI. Anomalies were calculated by differencing each identified TWI day from the average of the same calendar days calculated between 1981 and 2010.

**Figure 11:**
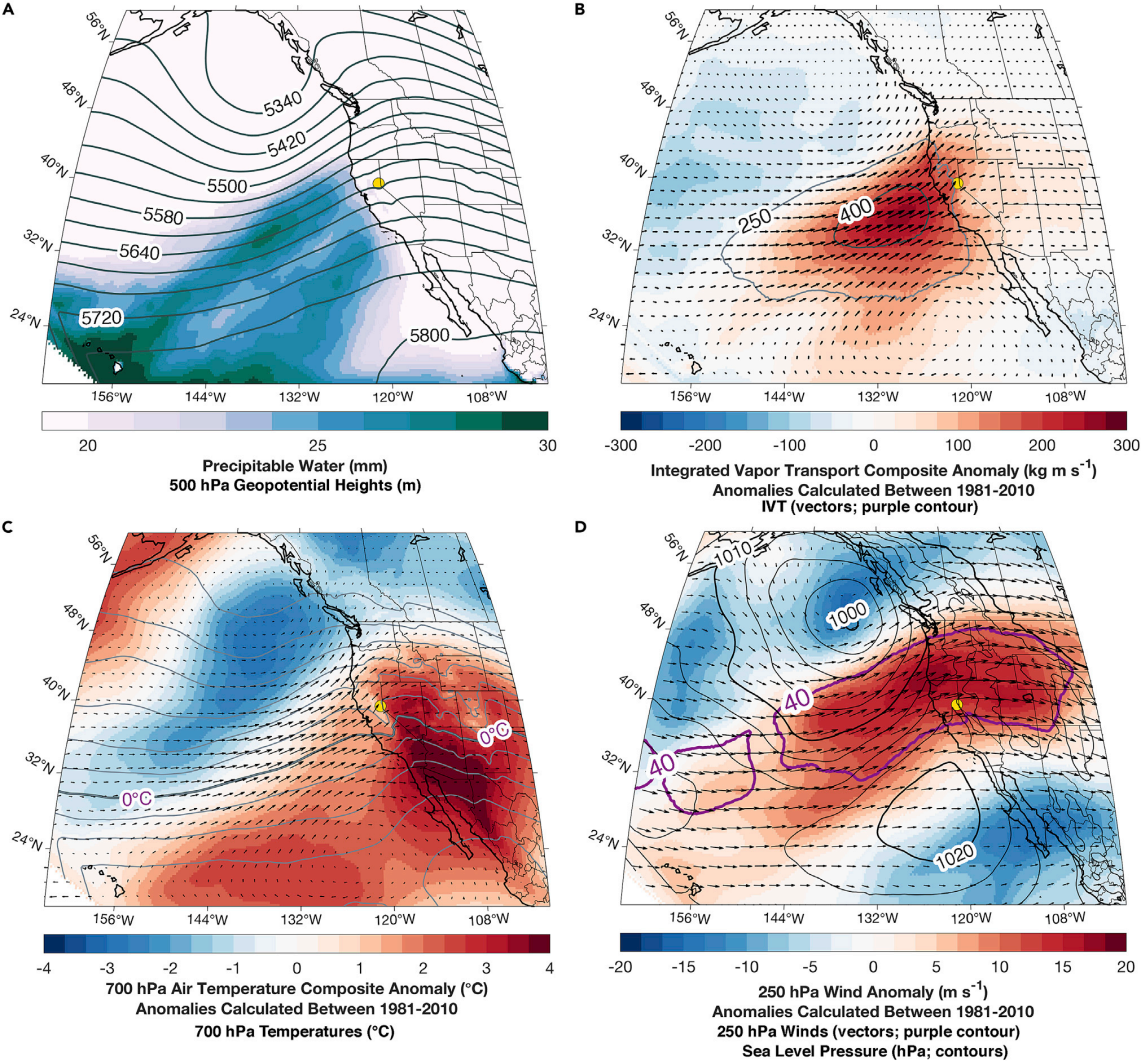
Composite synoptic conditions for events with at least six hours of TWI from ROS Composite synoptic conditions from the North American Regional Reanalysis (Mesinger et al., 2006) for 17 unique events that produced at least six hours of TWI. (A) Composite precipitable water (mm) and 500 hPa geopotential heights (m; contours). (B) Integrated vapor transport (IVT; kg m ^−1^ s ^−1^; relative vectors); IVT anomalies (colored); and regions indicating atmospheric river conditions (> 250 kg m^−1^ s ^−1^) or elevated moisture transport (> 400 kg m^−1^ s ^−1^). (C) 700 hPa air temperatures (contours) and 700 hPa air temperature anomalies (°C; filled contours) with IVT vectors overlaid (kg m^−1^ s ^−1^). (D) 250 hPa winds (vectors; m s ^−1^); 250 hPa wind anomalies (m s ^−1^; filled contours); 250 hPa winds exceeding 40 m s ^−1^ (purple contours); sea level pressure (hPa; black contours).

The presence of an offshore trough at 500 hPa and a broad plume of precipitable water (integrated water vapor) oriented in a southwest-northeast direction (Figure 11A) in conjunction with strong moisture flux (Figure 11B) and anomalously warm mountain-top (700 hPa temperatures; (Figure 11C)) are key components of storms producing heavy precipitation and high elevation snow levels in the Sierra Nevada (O’Hara et al., 2009; Kaplan et al., 2009; Hatchett et al., 2017a). The broad plume of precipitable water (Figure 11A) and integrated vapor transport in exceedance of 250 kg m ^−1^ s ^−1^ (Figure 11B) originating from the subtropics and extending northeastward into California are consistent with the typical genesis location of costly flood-producing atmospheric rivers (Prince et al., 2021). The dual composite moisture plumes indicate two primary corridors along which moisture export from the midlatitude cycle occurs leaving behind the footprint of concentrated water vapor (Dacre et al., 2015). The sustained liquid precipitation at CSSL needed to generate TWI is consistent with the strong moisture transport created by a baroclinic environment with anomalously cold air to the north and anomalously warm air to the south (Figure 11C). The sea level pressure gradient between lower pressure in the Gulf of Alaska and higher pressure off the coast of Baja California (Figure 11D) favors southwesterly winds blowing perpendicular to the Sierra Nevada and enhancing orographic uplift. The sustained precipitation at CSSL is further enhanced by quasigeostrophic ascent occurring in the exit region of the broader 500 hPa trough (Figure 11A). The 0°C isotherm at 700 hPa is located just south of CSSL (11C), suggesting mountain-top temperatures during TWI events on average are near-to-slightly above freezing, leading to greater fractions of precipitation falling as rain. Favored by a poleward shifted and anticyclonically curved upper level jet (Figure 11D), the anomalous warm temperatures (Figure 11C) indicate strong warm air advection and downstream geopotential height building as latent heat is advected into the region via transport of moist subtropical air (Figures 11A and 11B). These conditions are all broadly consistent with established synoptic patterns favoring heavy and sustained precipitation with elevated rain-snow transition elevations producing ROS and subsequent flooding (Hatchett et al., 2016, 2017a, 2020; Kaplan et al., 2009; O’Hara et al., 2009). This information provides additional insight to the snowpack runoff decision support in two ways. First, a forecast storm with some or all of these characteristics could prime the existing snowpack to actively produce runoff in a subsequent event by establishing preferential flow paths or reducing cold content (Brandt et al., 2022a). Second, regardless of the initial state of the snowpack, a forecast storm with these characteristics should elevate situational awareness for the potential to produce typical winter storm impacts in addition to TWI and subsequent runoff.

Our findings from the CSSL analysis suggest concerns about potential flooding should grow if more than 22 mm of precipitation as rain at the CSSL elevation is forecast for a 24 h period. By using higher confidence data via the quality-controlled hourly data and the TWI identification algorithm from more stations to identify historic circulation patterns to assess the potential for TWI, we both enhance confidence in our ability to capture impactful storms but also move further toward integrating an ingredients-based forecasting approach (e.g. Please adjust parenthesis: Doswell et al., (1996)) into the snowpack runoff decision support framework.

### Snowpack response to ROS

Our results, which show that midwinter storms can produce TWI from the snowpack while SWE increases (Figure 6), contradicts the previously held assumption that the snowpack has three separate time periods for warming (must become isothermal), ripening (maximum liquid water retention), and output (liquid water release) (Kinar and Pomeroy, 2015). Studies have documented that liquid water can move through the snowpack through the formation of preferential flow paths ahead of the wetting front though the dynamic feedback between present weather and snowpack conditions is not well understood (Marsh, 1999; Kattelmann and Dozier, 1999; Eiriksson et al., 2013; Jennings and Jones, 2015; Brandt et al., 2022a). For example, Berman et al. (2009) found isotope signatures transitioned from rain to snow, demonstrating the different time travel for rain water that only needed to remain warm enough to progress as liquid water. In contrast, snowmelt requires sufficient warming for the latent heat exchange within the snowpack. Consistent with these findings, hourly observations made at the CSSL SNOTEL provide evidence that the snowpack can release water while simultaneously accumulating SWE and increasing in density during ROS events. This implies that ROS does not always lead to a decrease in SWE (Guan et al., 2016). Furthermore, the hourly data demonstrated that liquid water content could increase as the snowpack charged with water and subsequently drained from the snowpack, similar to a rising and falling limb of a hydrograph, but with a positive net change in SWE. Therefore, decreases in SWE are not always synonymous with snowmelt during ROS events (Fassnacht and Records, 2015; Musselman et al., 2017, 2021; Yan et al., 2018; Henn et al., 2020; Brandt et al., 2022b). This highlights the value of using soil moisture as an indicator of TWI and that TWI should be used in tandem with other parameters to improve the definition of snowmelt in research, especially in ROS-prone regions.

### Value of the snowpack runoff decision support

With additional confidence in hourly data, we extracted information about present weather and antecedent snowpack conditions that coincided with midwinter TWI. This facilitated the development of the first iteration of a decision support framework indicating favorable snowpack runoff conditions for producing TWI (Figure 8). Leveraging observations to identify potential hazards is the first step to build reliance through impact-based decision support for a Weather Ready Nation (Uccellini and Ten Hoeve, 2019). Advisories and early warning systems represent a proven technique for communicating hazards to user communities. Operational examples in the United States are commonly provided by Federal agencies intending to provide decision support to regional user groups. Some examples include United States Forest Service avalanche forecasts (Statham et al., 2010), Environmental Protection Agency air quality index forecasts (Agency, 1999), U.S. Geological Survey post-fire debris flows assessments (Force, 2005), and the National Weather Service heat risk tool (Hawkins et al., 2017). Inspired by these advisory systems, and notably by the current lack of such a framework (to our knowledge) despite the known challenges and impacts for water management created by ROS in the western United States (e.g., McCabe et al. (2007); Musselman et al. (2018); Siirila-Woodburn et al. (2021)), we created this initial iteration of snowpack runoff decision support. We employed similar visualization strategies (e.g., color-coding following an ordinal scale) for communicating levels of runoff hazard. Similar to decision support developed to track the location of landfall and intensity of atmospheric rivers (Ralph et al., 2019), identifying regions with varying levels of risk for runoff based on antecedent snowpack conditions could increase the lead time for decision making and confidence in choices made. Coupling observation that provides information about the current state of the snowpack with an improved understanding of ROS processes (e.g. 22 mm of rainfall in 24 h at the CSSL always resulted in TWI) could provide more nuance to meteorological forecasts by better understanding the range of potential hydrological impacts ahead of the event. Another example includes forecast-informed reservoir operations, which are demonstrating the value of shifting from fixed flood control schedules toward risk-based ensemble forecasting to plan water releases (Delaney et al., 2020). A risk-based approach of a snowpack runoff decision support could be a valuable tool integrated into next-generation water resource management in transitional and snow-dominated regions. The use of hourly data highlights this advisory is possible and ongoing research aims to continue to develop the concept and address limitations as they are exposed through soliciting stakeholder feedback.

### Future work

This research aimed to identify present weather and antecedent snowpack patterns during midwinter TWI. However, a decision support tool is only beneficial when the information provided can reliably identify high-risk markers. Ongoing research will examine similar present weather and antecedent conditions that did not produce runoff. Understanding the constraints of TWI is important in order to disseminate low risk-markers to decision makers that need to conserve water resources without increasing flood risk.

Future research should integrate additional SNOTEL stations to test the robustness of the approach developed using the CSSL at a regional scale. Across the western US, midwinter snowmelt is increasing (Musselman et al., 2021) and the methods in this work can immediately be applied to identify midwinter snowmelt drivers as either ROS or warm day melt. Future work must refine the classification of antecedent snowpack conditions and storm characteristics and provide a linkage to streamflow responses to improve the prediction of runoff generation within a basin. The optimal result is dual-use data for real-time situational awareness of rapid changing snowpack conditions that overcome the capabilities of models and retrospective integration of high quality observational data for model validation and improve pattern recognition. Producing a quality-controlled hourly dataset can further the understanding of event-based snowpack dynamics, which could be valuable for forecast-informed reservoir operations (Yuba and Steering, 2021), flood management (Hatchett et al., 2016), landslide early warning systems (Baum and Godt, 2009), avalanche warning (Statham et al., 2010; Hatchett et al., 2017b), and design criteria for infrastructure such as culverts, levees, bridges, and reservoirs (Verdhen, 2018). By identifying TWI drivers and conditions leading to the greatest hydrometeorological impacts, we can develop a process-based tool to improve hydrological forecast confidence at longer lead times with the integration of meteorological forecasts through improved decision support (Uccellini and Ten Hoeve, 2019; Hatchett et al., 2020) that both improves protection of life and property and optimizes increasingly scarce water resources.

### Limitations of the study

While we have provided the initial steps toward operational snowpack runoff decision support, this study is limited in scope by only investigating one station, resulting in thresholds specific to the CSSL that have not yet been tested regionally. Caution should be exercised before the model and conceptual framework established at the CSSL is applied to other stations. However, the methods developed in this study can be applied to other SNOTEL stations and provide a testable framework to continue the research toward snowpack runoff decision support. Our approach would benefit greatly by subsequent engagement and iterative development activities in tandem with water management or other natural hazard-focused stakeholders as well as the incorporation of additional stations and data including, but not limited to streamflow, wind speed, solar radiation, and relative humidity for validation or to refine thresholds. Lessons learned and useful strategies from the development forecast-informed reservoir operations may facilitate this threshold-refining process.

The TWI identification algorithm will likely require calibration at other stations and will not work at stations without well-draining soil where soil is saturated throughout the winter. The algorithm developed for this study was intended to identify prominent periods of TWI but could not capture small increases in soil moisture without also capturing normal fluctuations during other periods not related to TWI.

Identifying each hour of TWI during ROS shows 6-h precipitation and temperature values that can be misleading. Once a precipitation event ends, the rolling 6-h precipitation total slowly decreases, but the snowpack could still release water. Some rainfall events turn to snow with cold frontal passage and erroneously associate the TWI with precipitation falling as snow (e.g. February 13–14, 2019). Precipitation phase classification from either *in situ* instrumentation such as disdrometers (Sumargo et al., 2020) or citizen science (Arienzo et al., 2021), would help further constrain ROS magnitude, TWI classification, and indicators of potential TWI.

### Conclusions

The motivation for our work was to investigate potential value that hourly data has to (1) improve process-based understanding of midwinter runoff generation and (2) provide real-time information to decision makers when rapid changes in the snowpack overcome the capability of the model. The SNOTEL network provides near real-time information valuable to the analysis of midwinter runoff and exceptional events, like the initiation and duration of ROS TWI from soil moisture sensors. These observations can be leveraged to develop a snowpack runoff decision support system by connecting observations to potential outcomes in order to mitigate risk (Uccellini and Ten Hoeve, 2019). We found value in the QA/QC hourly observations from a SNOTEL station, as these data can be used as input to decision support tools for pattern recognition and improve model accuracy by up to 25.7%. We then showed how this data can be applied to ingredients-based forecasting (Doswell et al., 1996) and could help to establish the framework for a regionally specific snowpack runoff decision support. In addition, our approach provided quantitative values of liquid precipitation required to produce a soil moisture response. Without a soil moisture response, runoff and subsequent flooding is unlikely. We also showed consistency between atmospheric conditions and identified ROS events using our framework, which provides additional confidence in the skill of the approach at correctly identifying physically consistent events.

Our efforts here represent a first step toward operational snowpack runoff decision support that is applicable across management scales and adjustable depending on flood management infrastructure. With increasingly frequent ROS and decreasing water availability projected in a warming climate (Musselman et al., 2018; Siirila-Woodburn et al., 2021), runoff advisories may become valuable tools to inform decision support for adaptive water management strategies such as forecast-informed reservoir operations (Delaney et al., 2020) or managed aquifer recharge (Steinschneider and Brown, 2012) intended to better capture and retain water to meet consumptive and ecosystem demands. By continuing to integrate the initial snowpack runoff decision support framework system with existing long-term hydrometeorological observational networks (e.g., Hatchett et al. (2020)) and by incorporating feedback from water managers, our approach can be continuously developed to provide increasingly useful impact-based decision support (Uccellini and Ten Hoeve, 2019) information in snow-dominated watersheds where water is managed as both a hazard and a resource.

## STAR★Methods

### Key resources table

**Table:** 

REAGENT or RESOURCE	SOURCE	IDENTIFIER
**Deposited data**		

Quality controlled hourly data	This Paper	https://github.com/aeheggli/SRAmethods
Manually classified terrestrial water input	This Paper	https://github.com/aeheggli/SRAmethods/blob/main/df_ML_ROSidentified.csv

**Software and algorithms**		

Python	Python Software Foundation	https://www.python.org/
Decision Tree Classifier	SciKit-Learn	https://scikit-learn.org/
Automated quality control and quality assurance	This Paper	https://github.com/aeheggli/SRAmethods/blob/main/CSSL_AutomatedQC.py
Manual quality control procedures	This Paper	https://github.com/aeheggli/SRAmethods/blob/main/CSSL_ManualQC.py
Decision tree classification (clean data)	This Paper	https://github.com/aeheggli/SRAmethods/blob/main/R2_DecisionTree_CSSL_QC.py
Decision tree classification (raw data)	This Paper	https://github.com/aeheggli/SRAmethods/blob/main/R2_DecisionTree_CSSL_Raw.py

**Other**		

SNOTEL hourly and daily data	NRCS Report Generator Data Search Tool	https://wcc.sc.egov.usda.gov/reportGenerator/
10-minute Geonor Precipitation gauge data	Desert Research Institute Western Regional Climate Center	https://wrcc.dri.edu/cgi-bin/rawMAIN.pl?cacssl
Daily manual observations	University of California Central Sierra Snow Laboratory	https://doi.org/10.6078/D1941T

### Resource availability

#### Lead contact

Further information and requests for resources should be directed to and will be fulfilled by the lead contact, Anne Heggli (anne.heggli@dri.edu or aeheggli@gmail.com).

#### Materials availability

Not applicable.

#### Data and code availability

• Comparative data from the Central Sierra Snow Lab (CSSL) was accessed from three locations for water years 2006–2019:
• NRCS Report Generator Data Search Tool: https://wcc.sc.egov.usda.gov/reportGenerator/
• Desert Research Institute’s Western Regional Climate Center: https://wrcc.dri.edu/cgi-bin/rawMAIN.pl?cacssl
• University of California Central Sierra Snow Laboratory: https://cssl.berkeley.edu/
• The data files used in these methods have been deposited to https://github.com/aeheggli/SRAdata. These data are publicly available as of the date of publication.
• Quality Controlled and Quality Assured hourly SNOTEL data generated in this study have been deposited to https://github.com/aeheggli/SRAmethods and are publicly available as of the date of publication.
• Code used for the first step of automated quality control and quality assurance has been deposited to https://github.com/aeheggli/SRAmethods/blob/main/CSSL_AutomatedQC.py and is publicly available as of the date of publication.
• Code used for the manual quality control procedures has been deposited to https://github.com/aeheggli/SRAmethods/blob/main/CSSL_ManualQC.py and is publicly available as of the date of publication.
• Code used for the decision tree classification has been deposited to https://github.com/aeheggli/SRAmethods/blob/main/DecisionTree_CSSL_QC.py and https://github.com/aeheggli/SRAmethods/blob/main/DecisionTree_CSSL_raw.py and is publicly available as of the date of publication.
• Manually classified terrestrial water input data has been deposited to https://github.com/aeheggli/SRAmethods/blob/main/df_ML_ROSidentified.csv and is publicly available as of the date of publication.
• Any additional information required to reanalyze the data reported in this paper is available from the lead contact upon request.

### Experimental model and subject details

Not applicable.

### Method details

#### Study location

Our study location is the University of California, Berkeley Central Sierra Snow Laboratory (CSSL; 2). The CSSL was established in 1946 and is currently co-located with a US Department of Agriculture Natural Resource Conservation Service (NRCS) SNOw TELemetry (SNOTEL) station at 2,100 m elevation approximately 2 km west of the Sierra Nevada crest in Soda Springs, California (Figure 2). The CSSL SNOTEL station (#428) began collecting data in 1982 with a precipitation gauge, a snow pillow to monitor snow water equivalent (SWE), and an air temperature sensor. In 2005, the station was upgraded with an ultrasonic snow depth sensor and soil moisture and temperature sensors at 5, 20, and 50 cm depths (https://wcc.sc.egov.usda.gov/nwcc/site?sitenum=428). The CSSL provides a unique opportunity to inform conditions in three highly managed watersheds in California and Nevada. While it sits in the headwaters of the westward-draining South Fork of the Yuba River, it is 3 km north of the westward-draining North Fork of the American River and 2 km east of the headwaters of the eastward-draining Truckee River watershed. The terrain surrounding the CSSL is predominantly exposed Jurassic to Cretaceous granitic bedrock overlaid with tertiary volcanic deposits. The surrounding forest is comprised of Lodge Pole Pine *(Pinus murrayana*), Red Fir (*Abies magnifica*), and Whitebark Pine (*Pinus albicaulis*) with timberline occurring at approximately 2,500 m (Osterhuber, 2009). Using the Köppen climate classification system, the CSSL experiences a Humid Continental Climate with a Dry Cool Summer (Dsb). The region west of the crest is characterized by a Warm Summer Mediterranean climate (Csb) while the region to the east is characterized by a Cold Desert Climate (BWk).

Data for this study was selected for water year 2006, when the SNOTEL station was upgraded, through 2019. Water years 2011 and 2012 were not included in the analysis because the 20 cm soil moisture sensor stopped reporting. The median annual precipitation for the period of study was 1,576 mm and median maximum SWE was 946 mm. The period of study captures the highest (2017) and second lowest (2014) precipitation totals as well as the second largest (2017) and lowest SWE totals since 1983 when the SNOTEL record began. Additionally, there were several significant ROS events within this period that produced floods in the region (Hatchett et al., 2016). The most notable of these were January 2006 and February 2017 (Underwood et al., 2009; Henn et al., 2020).

#### Instrumentation

This section intends to provide the reader with an understanding of the instrumentation used to collect data and the limitations of each. An important aspect of quality control procedures is understanding limitations and functionality of the instrumentation. For example, a tipping bucket rain gauge, an all-weather storage gauge, and a weighing precipitation gauge all experience different data issues, and each requires unique quality control procedures to address those even though all measure precipitation.

#### Snow water equivalent

The CSSL SNOwpack TELemetry (SNOTEL) station monitors snow water equivalent (SWE) with a four panel 1.2 × 1.5 m stainless steel snow pillow array. Snow pillows measure applied weight; complex snowpack structures including crust layers and changes in applied weight caused by snow creep and thermal differences at the snow-sensor-soil interface can cause over and under reading (Serreze et al., 1999; Johnson and Schaefer, 2002; Johnson, 2004; Johnson and Marks, 2004; Julander, 2007). Snow pillow data errors include over- and under-reading, temperature-impacted diurnal flutter, and bladder leaks causing a steady decrease in SWE.

Manual SWE observations are taken at the discretion of the CSSL staff with a Federal sampler or from a snow pit. Federal sampler measurements typically over measure in deep snowpacks by 7–12% and can under-measure in lower density snow or when depth hoar is present (Work et al., 1965; Peterson and Brown, 1975; Farnes et al., 1980, 1982). The accuracy of the measurement is impacted by the skill of the snow surveyor and by the snowpack conditions. The Federal sampler is well-correlated (R = 0.94) when analyzed against snow pillows with a correlation of 0.98 for the period of study at the CSSL (Cox et al., 1978).

#### Precipitation

The SNOTEL automated all-season storage gauge measures the volume of water via a pressure transducer that measures the change in hydro-static pressure in the column of the collector. The SNOTEL gauge is 7.3 m tall, has a 730 cm^2^ orifice, and has an Alter shield. During snowfall events snow can collect on the inside of the catch-can causing a ”plug” in the gauge (Goodison et al., 1998; McGurk, 1986). Diurnal temperature swings can cause the fluid and the SNOTEL gauge itself to expand and contract, resulting in a change in hydro-static pressure applied to the pressure transducer and negative precipitation values that interfere with data analysis (NRCS, 2014).

The Geonor T-200B is a weighing precipitation gauge with a vibrating wire that changes frequency as precipitation is collected. The T-200B has a 200 cm^2^ orifice and an Alter shield installed. The T-200B measures the change in vibrating wire frequency, which is related to the precipitation accumulated, not the hydrostatic pressure. Diurnal flutter could still slightly impact the observations made by the T-200B, but none were identified during this project. The Geonor T-200B is the preferred gauge for the CSSL manual observations, but manual precipitation observations were made at the discretion of the CSSL manager. Deviations in manual observations from the T-200B data appears to indicate time periods where the Geonor sensor may have had issues.

#### Snow depth

SNOTEL stations utilize Judd ultrasonic snow depth sensors, which are acoustic sensors that emit a sound wave and measure the travel time with an integrated retry algorithm as an attempt to minimize the data issues during precipitation events (Anderson and Wirt, 2008). When conditions prevent a sensor from making a measurement, the sensor retries 10 times before outputting a full-scale value. Snowpack observations with ultrasonic sensors include two main issues: (1) diurnal fluctuation due to the dependence on temperature at the sensor and throughout the column of air and (2) inaccurate readings during precipitation events from the reflection from falling hydrometeors rather than the snowpack surface (Hauptmann et al., 2002). However, once the snow accumulation season starts and between snow storms, the ultrasonic snow depth sensors obtain reliable measurements when properly maintained (NRCS, 2014). The metadata for the ultrasonic snow depth sensor at the CSSL SNOTEL verified regular maintenance from when the Judd sensor was installed in 2005 and replaced in 2012 and 2017.

Snow depth observations are made daily at 9 am local Pacific time (1700 or 1800 UTC, depending on time of year) from the snow stake. The snow stake is approximately 35 m from the location of the SWE measurements. The manual snow depth observations were not used with manual SWE observations to calculate density since they are not taken from the same location. Ultrasonic snow depth sensors and manually observed snow depth typically have high correlation of 0.96–0.99 (Anderson and Wirt, 2008; Bergman, 1989; Goodison et al., 1998). The CSSL has high correlation of 0.989 between SNOTEL daily QC product and the manually observed snow depth from snow stake for the period of this study. This demonstrates the utility of daily snow depth data to verify magnitudes of events, but not to correct absolute values.

#### Air temperature and soil moisture

An extended range YSI temperature sensor was installed in 2005. A calibration concern was discovered for the SNOTEL air temperature sensors and a correction is required (Brown et al., 2019). The air temperature data has been corrected with the preliminary equation to be updated when the NRCS completes the final review. There were no further quality control procedures required. The NRCS monitors soil moisture and temperature at 5, 20, and 50 cm depths with a Steven’s HydroProbe. Soil moisture data for this period of study was independently quality controlled at the hourly time-step by the NRCS, and therefor did not require additional quality control procedures or editing for this study (Sutcliffe and Clayton, 2021).

#### Quality control (QC) and quality assurance (QA) methods

Data was acquired from publicly available sources and can be located in the key resources table. Hourly SNOTEL data was used for all parameters as the dataset of focus for the application of QA/QC methods. The proposed QC procedure for SWE, precipitation, and snow depth consists of three levels: Level 1: a range check; Level 2: an inter-sensor comparison; and Level 3: using human expert judgement (Kondragunta and Shrestha, 2006; Oakley et al., 2018). Auxiliary data was downloaded to use for Level 2 QC. SNOTEL daily data is subject to quality control procedures outlined in detail in the National Engineering Handbook Part 622: Snow Survey and Water Supply Forecasting (NRCS, 2014). Daily observations made by the station manager at the Central Sierra Snow Lab and 10-min precipitation data from the WRCC were also collected for Level 2 QC.

The NRCS QA and QC flagging system was adapted for the development of these methods (Table 2). QA flags designate the level of review that the data has passed: Raw (R) for data that has not undergone any review, Flagged (F) to identify data that passed the automated data flagging, Provisional (P) for data that has undergone preliminary human review, and Approved (A) indicating that a final review was completed to archive the data. QC flags indicate if any measures were taken to improve the quality of the data. Three of the NRCS QC flags were applied to these methods: Valid (V) for data that passed preliminary QC checks, Edited (E) for data that was edited, and Suspect (S) for data that does not pass QC checks, requires further review, or cannot be edited to reliably improve the quality of the data.

**Table:** QA and QC flagging system adapted from NRCS Snow Survey and Water Supply Forecasting National Engineering Handbook

Quality Assurance (QA) Flags		
R	Raw	No Human Review
F	Flagged	Automated QC Flag Assigned
P	Provisional	Preliminary Human Review
A	Approved	Processing and Final Review Completed
**Quality Control (QC) Flags**		
Flag	Name	Description
V	Valid	Validated data
E	Edit	Edit existing value
S	Suspect	Suspect data

Data was corrected with a semi-automated approach developed by A. Heggli (Python code available from the repository information) to limit subjective editing and encourage a more repeatable workflow that directs the data reviewer to the sensor or time periods that require human review. The Level 1 QC range check values were identified using the NRCS station profiles that outline maximum and minimum daily values and maximum and minimum daily changes for each observation type. Precipitation, snow water equivalent, and snow depth sensors are impacted by temperature changes that result in diurnal flutter in the data. Rolling median calculations for 6, 12, or 24-h windows are used as guides to minimize diurnal flutter and outlined in detail by observation type in Value of the snowpack runoff decision support section. Once the automated QA/QC procedure was completed, the data was saved as a separate preliminary file for the second phase of QA/QC using Level 2 (inter sensor comparison) and Level 3 (human-expert judgment). Preliminary data was manually reviewed using the CSSL_ManualQC code, which provides interactive visualization to aid in the manual editing of Excel files saved as .csv format file. Rolling median calculations mute peaks and delay the signal response, therefore events were manually retained, and calculations were applied so the automated guide was centered on the observation and any slight delay was considered negligible on an hourly time step (Figure S1). If data quality could not be verified, then raw data was retained and flagged as suspect. Once all parameters had gone through full preliminary review, the QA flags were changed to P (provisional) and saved as a separate provisional file. The QA flag can be updated to A once the reviewer is confident that the data has attained the highest accuracy possible. The QC flags will be retained as indication of the quality of the data. Level 2 and Level 3 QC procedures are sensor dependent and outlined independently for each observation in Future work section.

#### Quality control (QC) methods by observation type

##### Snow water equivalent

Data is first filtered with the QC Level 1 range check to identify outliers, which are then classified as suspect. Negative values are replaced with a zero and values over the maximum are set to null and flagged as suspect to be reviewed against the original data and edited as necessary. A 6-hr median was used as the guide to reduce diurnal noise. Hourly SWE data deviating from the daily QC product and not following the trend of the manual observations was reviewed against temperature, precipitation, and soil moisture data to either correct the data and retain it or set it as suspect. Bridging of the snow pillow was identified through inter-sensor comparison (Level 2 QC) with precipitation and temperature data for evidence of rain-on-snow (ROS) or a prolonged period of above freezing days followed by below freezing temperatures that could lead the formation of a crust layer prior to the erratic data from the pillow (Figure 3). Potential bridging was flagged as suspect.

Soil moisture was used to validate suspect SWE data (Figure 2A). Between February 12–15, 2019 an exceptionally strong atmospheric river made landfall in California (Hatchett et al., 2020) and impacted the CSSL (Figure 2B). Abrupt increases in soil moisture validates the SWE data, which responded to the ROS event by increasing 140 mm before decreases in SWE were observed. The snowpack began to release water approximately four hours after the precipitation occurred with temperatures above 0°C but 11 hours before the decline in SWE. This could be an indication of the formation of preferential flow paths since a uniform wetting front would result in SWE accumulating until the wetting front reaches the base of the snowpack at which point SWE would begin to plateau or decrease (Marsh and Woo, 1984). Peak SWE increases also correspond with increase of soil moisture as the snowpack transitions from the midwinter accumulation and ripening period to the ablation period (Figure 2C).

##### SWE QC summary

• Level 1 range check: Identify data outside of the bounds of the profile and set to suspect. Set negative values to zero and positive exceeding values as null.
• Level 2 inter-sensor comparison: SNOTEL Daily QC product - Check for deviation and set abnormal values to suspect.
• Level 2 inter-sensor comparison: Diurnal fluctuation - review against temperature.
• Level 2 inter-sensor comparison: Bridging - review against temperature and precipitation.
• Level 2 inter-sensor comparison: ROS signatures - review against precipitation, rainfall, and soil moisture.
• Level 2 inter-sensor comparison: Peak SWE jumps - review against temperature and soil moisture.
• Level 3 human expert judgement: Flag level 2 data accordingly. Review all ”S” flags to verify they were flagged appropriately. Use expert judgement to edit SWE data and change QC flag to ”E” or leave raw data values and retain the ”S” QC flag. Unedited data that passed the QC check is flagged as ”V”.

#### Precipitation quality control (QC) methods

After the Level 1 check, hourly SNOTEL data was verified against the SNOTEL daily QC product, WRCC Geonor data, and the CSSL observations. The Geonor weighing rain gauge and snow pillow were used to identify the initiation and termination of precipitation events to correct for snow plug formation and release. When the daily QC products showed no increase in precipitation, diurnal variation in data between events were removed manually.

The release of snow plugs is identified when there is an abnormal increase in the hourly data. A the CSSL precipitation of 10 mm/h or more should be manually reviewed to validate the precipitation event or correct for a snow plug. After snow plug releases were identified, hourly SWE data and the weighing rain gauge data were used to identify the initiation of the event. Since snow pillows can provide accurate precipitation data during snowfall, hourly increases of SWE were added to the precipitation values until the snow plug released and precipitation measurements resumed accurately from the SNOTEL station (Figure 3). The 24-h changes in precipitation were checked against the SNOTEL daily QC product and the manual observations.

##### Precipitation QC summary

• Level 1 range check: Identify all hourly data outside of the bounds of the profile and set to suspect. Set negative values to zero and positive exceeding values as null.
• Level 2 inter-sensor comparison: Diurnal flutter - review against daily QC product to eliminate diurnal variation when no precipitation occurs.
• Level 2 inter-sensor comparison: Snow plugs - review against snow pillow and daily QC precipitation product to verify plug formation.
• Level 3 human expert judgement: Review all ”S” flags to verify they were flagged appropriately. Use expert judgement to refer to snow pillow data to fill data until the snow plug releases. If the storm starts as rain and turns to snow, use temperature data as a reference of when it was likely that the plug began to form. Edit data and change QC flag to ”E” or leave raw data values and retain the ”S” QC flag. Unedited data that passed the QC check is flagged as ”V”.

#### Snow depth quality control (QC) methods

Snow depth data first was subjected to Level 1 QC range check. Suspect values that were verified to be full-scale readings during snowfall events were set to null and linear interpolation was used to fill in values. To reduce the flutter in the data, a 6-hr and 12-hr rolling median was used as a guide for snow accumulation periods, the 24-hr rolling median was used as a guide during compaction, and peak snowpack was manually retained (Figure 1). The SNOTEL daily QC snow depth values were used to verify edited values. The CSSL snow depth observations were used to verify changes in snow depth and the general trend of the data throughout the water year. The final check was to make sure SWE and snow depth reached zero on the same day.

##### Snow depth QC summary

• Level 1 range check: Identify all hourly data outside of the bounds of the profile and set to suspect. Set negative values to zero and positive exceeding values as null.
• Level 2 inter-sensor comparison: Missing data - verify full-scale readings occurred during snowfall events by reviewing precipitation, SWE, and temperature changes. Apply a linear interpolation.
• Level 2 inter-sensor comparison: Diurnal flutter - review against temperature changes and apply rolling median to reduce temperature educed gains and losses.
• Level 3 human expert judgement: Review all ”S” flags to verify they were flagged appropriately. Use expert judgement to edit data and change QC flag to ”E” or leave raw data values and retain the ”S” QC flag.

#### Terrestrial water input (TWI) identification algorithm

Terrestrial water input (TWI) is water input to the land surface from either precipitation or melting snow. TWI can be identified from shallow soil moisture observations ((Flint et al., 2008; Sutcliffe and Clayton, 2021). TWI was identified when soil moisture increased 0.5% in one hour or 1.0% in two hours at 5 cm and 20 cm depths. The soil moisture data has a resolution of 0.1%; a threshold of 0.4% identified 200 additional TWI events as a result of normal variation in the sensor. A 0.6% increase over one hour identified 30 less TWI events, so the 0.5% threshold was selected to capture the majority of events while reducing misidentified TWI events triggered by noise. A threshold of 1.0% aided in identifying events that had a slower initial increase while maintaining the same rate of change. Saturation was identified through observations of soil moisture values during spring snowmelt where each sensor asymptotes at a value of 39%. TWI was classified using the rate of change identification parameters or when all three sensors were saturated. The soil at the 5 and 20 cm depths at the CSSL is well-draining and soil moisture begins to recede when TWI ceases. The TWI identification algorithm will likely require calibration for other locations due to site specifics like soil properties.

### Quantification and statistical analysis

#### Decision tree classification

The Decision Tree Classifier is a supervised machine learning algorithm selected to aid in pattern recognition of midwinter snowpack TWI drivers (Pedregosa et al., 2011). The decision tree can handle continuous and categorical data, does not require the normalization or scaling of data, and can automatically handle missing values. Decision trees present a series of questions that split data into branches using the Gini Impurity where a value of 0 is a pure classification split and 0.5 is an impure split that incorrectly classified half of the samples.

The decision process begins by identifying the initial ro node by calculating the weighted sum of the Gini Impurity from all the possible sub-nodes. This is repeated on each impure internal node creating branches until the tree is complete with only pure leaf nodes. Growing the tree until all pure leaf nodes are achieved often causes over-fitting of the model. Decision trees are sensitive to noise in the data (Pedregosa et al., 2011) meaning small changes to the data can result in large changes to the structure of the tree. The learning process of the Decision Tree Classifier was designed with these limitations in mind. The maximum depth of the tree is limited to reduce over-fitting, data was hand-cleaned to reduced noise and increase stability, and the application of the tree as an aid in pattern recognition allows flexibility to address any changes in the tree structure. The Python code for the clean and raw data can be found in the repository information in Data and code availability section.

#### Feature engineering

There are a total of four native features in the hand cleaned data: precipitation, SWE, snow depth, and air temperature. Soil moisture at all three depths were used to develop the TWI target variable and therefore not included in feature engineering or feature selection. Machine learning algorithms compare data from a single point in time. However, the evolution and state of the snowpack is dependent on weather, which is transient by nature. Features were engineered to include information from the current time for up to 12 hours before TWI was identified. To identify TWI related to present weather, the following features were engineered: 1–6 h precipitation totals, 12 h precipitation total, and 6 h maximum temperature.

#### Filtering data for midwinter snow-cover

Midwinter is defined in this paper to include snow cover when SWE was greater than 100 mm before the ablation period melt begins following peak SWE (median date 24 March). Shallow snow, defined in this study when SWE is less than 100 mm, requires less energy input than deeper snow to melt and initiate runoff and therefore considered to be perpetually at higher risk of melting (Berris and Harr, 1987; Colbeck, 1976; Harr, 1981; Marks et al., 1998, 2001).

SWE at the CSSL does not always follow a typical SWE accumulation pattern with a defined peak leading into the ablation period. Some water years (WY), such as WY2013, present a plateau before the initiation of the spring ablation period while other years, such as WY2014, display two peaks (Figure 5). Midwinter ablation periods identified in 2014 and 2015 were filtered out of the training data for this study. Data qualified as suspect during the QC process were also filtered out of the training data.

#### Target variable: ROS or warm day melt TWI

The TWI driver was manually identified at each data point through inter-sensor comparison and human expert judgement (Kondragunta and Shrestha, 2006) and is available in the repository information in Data and code availability section. Warm day melt was assigned a value of 0. Warm day melt was manually identified when there was no recent precipitation, a maximum daily temperature that peaked above at least 5°C with TWI typically initiating in the late afternoon (around 13:00 local time) and stopping in the evening (around 17:00 local time). This TWI cycle follows the lag of diurnal temperature and is consistent with expected the outcome of warm day melt. ROS was assigned a value of 1 if there was active or recent precipitation that corresponded with above freezing temperatures. It is not yet possible to identify TWI drivers with numeric thresholds as changes in snowpack depth, density, and prior wetting can inhibit TWI under some meteorological conditions but not others; identifying these patterns is one of the objectives of this study.

#### Decision tree classifier criteria

The decision tree classifier was trained on data from WYs 2008–2019 and tested against WYs 2006 (wet year; four events) and 2007 (dry year; two events). WY2011 and WY2012 were missing soil moisture data and not included in the training data. The manually identified TWI driver was used as the target variable. A maximum depth of the decision tree was set to four to assure the model did not over fit and provide a comprehensible tree. The model was run using the clean data and the raw data to measure the model improvement achieved with cleaned data. A cross-validation of model performance was also performed for each set of two consecutive years (eg. 2008 & 2009, 2009 & 2010, 2010 & 2013, etc.) for both clean and raw data.

## References

[bib1] Agency E.P. (1999). 40 cfr part 58 air quality index reporting; final rule. Fed. Regist..

[bib2] Anderson E. (2006). Snow accumulation and ablation model-SNOW-17. Tech. Rep..

[bib3] Anderson J., Wirt J. (2008). Ultrasonic snow depth sensor accuracy, reliability, and performance, Hood River, OR. http://sites/westernsnowconference.org/PDFs/2008Anderson.pdf.

[bib4] Arienzo M.M., Collins M., Jennings K.S. (2021). Enhancing engagement of citizen scientists to monitor precipitation phase. Front. Earth Sci..

[bib5] Avanzi F., Michele C.D., Ghezzi A., Jommi C., Pepe M. (2014). A processing–modeling routine to use SNOTEL hourly data in snowpack dynamic models. Adv. Water Resour..

[bib6] Bales R.C., Hopmans J.W., O’Geen A.T., Meadows M., Hartsough P.C., Kirchner P., Hunsaker C.T., Beaudette D. (2011). Soil moisture response to snowmelt and rainfall in a sierra Nevada mixed-conifer forest. Vadose Zone J..

[bib7] Bartles M., Brauer T., David Ho M.F., Karlovits G., Pak J., Van N., Willis J. (2021). Hydrologic modeling system HEC-HMS User’s manual. Tech. Rep..

[bib8] Baum R.L., Godt J.W. (2009). Early warning of rainfall-induced shallow landslides and debris flows in the USA. Landslides.

[bib9] Bergman J.A. (1989). Proceedings of The Western Snow Conference.

[bib10] Berman E.S.F., Gupta M., Gabrielli C., Garland T., McDonnell J.J. (2009). High-frequency field-deployable isotope analyzer for hydrological applications. Water Resour. Res..

[bib11] Berris S.N., Harr R.D. (1987). Comparative snow accumulation and melt during rainfall in forested and clear-cut plots in the western cascades of Oregon. Water Resour. Res..

[bib13] Brandt W.T., Haleakala K., Hatchett B.J., Pan M. (2022). A review of the hydrologic response mechanisms during mountain rain-on-snow. Front. Earth Sci..

[bib12] Brandt W.T., Cannon F., Cooper A., Monache L.D., Haleakala K., Hatchett B.J., McGurk B., Pan M., Ralph F.M. (2022).

[bib14] Brown C.R., Domonkos B., Brosten T., DeMarco T., Rebentisch A. (2019). 87th Annual Western Snow Conference.

[bib15] Cardell-Oliver R., Kranz M., Smettem K., Mayer K. (2005). A reactive soil moisture sensor network: design and field evaluation. Int. J. Distributed Sensor Networks.

[bib16] Cayan D.R., Georgakakos K.P. (1995). Hydroclimatology of continental watersheds: 2. spatial analyses. Water Resour. Res..

[bib17] Chang E.K.M., Zheng C., Lanigan P., Yau A.M.W., Neelin J.D. (2015). Significant modulation of variability and projected change in California winter precipitation by extratropical cyclone activity. Geophys. Res. Lett..

[bib18] Chen X., Leung L.R., Wigmosta M., Richmond M. (2019). Impact of atmospheric rivers on surface hydrological processes in western u.s. watersheds. J. Geophys. Res. Atmospheres.

[bib19] Cho E., Jacobs J.M. (2020). Extreme value snow water equivalent and snowmelt for infrastructure design over the contiguous United States. Water Resour. Res..

[bib20] Church J. (1948). The evolution of snow-melt by dyes and drip-pan. Int. Assoc. Hydrological Sci. Gen. Assembly Oslo.

[bib21] Clark M.P., Nijssen B., Luce C.H. (2017). An analytical test case for snow models. Water Resour. Res..

[bib22] CNRFC (2021). Heavy precipitation event southwest Oregon, northern California, and western Nevada December 24, 2005 - january 3, 2006. Tech. Rep..

[bib23] Colbeck S.C. (1972). A theory of water percolation in snow. J. Glaciology.

[bib24] Colbeck S.C. (1976). An analysis of water flow in dry snow. Water Resour. Res..

[bib25] Cox L., Bartee L., Crook A., Farnes P., Smith J. (1978). Proceedings of the Western Snow Conference.

[bib26] Dacre H.F., Clark P.A., Martinez-Alvarado O., Stringer M.A., Lavers D.A. (2015). How do atmospheric rivers form?. Bull. Am. Meteorol. Soc..

[bib27] Delaney C.J., Hartman R.K., Mendoza J., Dettinger M., Monache L.D., Jasperse J., Ralph F.M., Talbot C., Brown J., Reynolds D., Evett S. (2020). Forecast informed reservoir operations using ensemble streamflow predictions for a multipurpose reservoir in northern California. Water Resour. Res..

[bib28] Dettinger M.D., Anderson M.L. (2015). Storage in California’s reservois and snowpack in this time of drought. San Francisco Estuary and Watershed. Science.

[bib29] Dettinger M.D., Cayan D.R. (1995). Large-scale atmospheric forcing of recent trends toward early snowmelt runoff in California. J. Clim..

[bib30] Doswell C.A., Brooks H.E., Maddox R.A. (1996). Flash flood forecasting: an ingredients-based methodology. Weather Forecast..

[bib31] Eiriksson D., Whitson M., Luce C.H., Marshall H.P., Bradford J., Benner S.G., Black T., Hetrick H., McNamara J.P. (2013). An evaluation of the hydrologic relevance of lateral flow in snow at hillslope and catchment scales. Hydrological Process..

[bib32] Farnes P.E., Goodison B.E., Peterson N.R., Richards R.P. (1980). Proposed metric snow samplers, proceedings of the western snow conference, Laramie, Wyoming. http://sites/westernsnowconference.org/PDFs/1980Farnes.pdf.

[bib33] Farnes P.E., Peterson N.R., Goodison B.E., Richards R.P. (1982). Metrication of manual snow sampling equipment by western snow conference metrication committee. Proc. Western Snow Conf..

[bib34] Fassnacht S.R., Records R.M. (2015). Large snowmelt versus rainfall events in the mountains. J. Geophys. Res. Atmospheres.

[bib35] Flint A.L., Flint L.E., Dettinger M.D. (2008). Modeling soil moisture processes and recharge under a melting snowpack. Vadose Zone J..

[bib36] Force N.U.D.F.T. (2005).

[bib37] Georgakakos K.P. (2006). Analytical results for operational flash flood guidance. J. Hydrol..

[bib38] Godsey S.E., Kirchner J.W., Tague C.L. (2014). Effects of changes in winter snowpacks on summer low flows: case studies in the sierra Nevada, California, USA. Hydrological Process..

[bib39] Goodison B.E., Louie P.Y., Yang D. (1998).

[bib40] Guan B., Waliser D.E., Ralph F.M., Fetzer E.J., Neiman P.J. (2016). Hydrometeorological characteristics of rain-on-snow events associated with atmospheric rivers. Geophys. Res. Lett..

[bib41] Harr R. (1981). Some characteristics and consequences of snowmelt during rainfall in western Oregon. J. Hydrol..

[bib42] Hatchett B., Daudert B., Garner C., Oakley N., Putnam A., White A. (2017). Winter snow level rise in the northern sierra Nevada from 2008 to 2017. Water.

[bib43] Hatchett B.J., Burak S., Rutz J.J., Oakley N.S., Bair E.H., Kaplan M.L. (2017). Avalanche fatalities during atmospheric river events in the western United States. J. Hydrometeorology.

[bib44] Hatchett B.J., Cao Q., Dawson P.B., Ellis C.J., Hecht C.W., Kawzenuk B., Lancaster J.T., Osborne T.C., Wilson A.M., Anderson M.L. (2020). Observations of an extreme atmospheric river storm with a diverse sensor network. Earth Space Sci..

[bib45] Hatchett B.J., Kaplan M.L., Burak S. (2016). Proceedings of the International Snow Science Workshop.

[bib46] Hatchett B.J., McEvoy D.J. (2018). Exploring the origins of snow drought in the northern sierra Nevada, California. Earth Interactions.

[bib47] Hauptmann P., Hoppe N., Püttmer A. (2002). Application of ultrasonic sensors in the process industry. Meas. Sci. Technology.

[bib48] Hawkins M.D., Brown V., Ferrell J. (2017). Assessment of noaa national weather service methods to warn for extreme heat events. Weather, Clim. Soc..

[bib49] He M., Whitin B., Hartman R., Henkel A., Fickenschers P., Staggs S., Morin A., Imgarten M., Haynes A., Russo M. (2016). Verification of ensemble water supply forecasts for sierra Nevada watersheds. Hydrology.

[bib50] Henn B., Musselman K.N., Lestak L., Ralph F.M., Molotch N.P. (2020). Extreme runoff generation from atmospheric river driven snowmelt during the 2017 oroville dam spillways incident. Geophys. Res. Lett..

[bib51] Hirashima H., Yamaguchi S., Sato A., Lehning M. (2010). Numerical modeling of liquid water movement through layered snow based on new measurements of the water retention curve. Cold Regions Sci. Technology.

[bib52] Jasechko S., Birks S.J., Gleeson T., Wada Y., Fawcett P.J., Sharp Z.D., McDonnell J.J., Welker J.M. (2014). The pronounced seasonality of global groundwater recharge. Water Resour. Res..

[bib53] Jennings K., Jones J.A. (2015). Precipitation-snowmelt timing and snowmelt augmentation of large peak flow events, western cascades, Oregon. Water Resour. Res..

[bib54] Johnson J.B. (2004). A theory of pressure sensor performance in snow. Hydrological Process..

[bib55] Johnson J.B., Marks D. (2004). The detection and correction of snow water equivalent pressure sensor errors. Hydrological Process..

[bib56] Johnson J.B., Schaefer G.L. (2002). The influence of thermal, hydrologic, and snow deformation mechanisms on snow water equivalent pressure sensor accuracy. Hydrological Process..

[bib57] Julander R.P. (2007). Soil surface temperature difference between steel and hypalon pillows. http://sites/westernsnowconference.org/PDFs/2007Julander.pdf.

[bib58] Julander R.P., Holcombe J. (2005). Proceedings of the Western Snow Conference.

[bib59] Kaplan M.L., Adaniya C.S., Marzette P.J., King K.C., Underwood S.J., Lewis J.M. (2009). The role of upstream midtropospheric circulations in the sierra Nevada enabling leeside (spillover) precipitation. part ii: a secondary atmospheric river accompanying a midlevel jet. J. Hydrometeorology.

[bib60] Katsushima T., Yamaguchi S., Kumakura T., Sato A. (2013). Experimental analysis of preferential flow in dry snowpack. Cold Regions Sci. Technology.

[bib61] Kattelmann R. (1985). Macropores in snowpacks of sierra Nevada. Ann. Glaciology.

[bib62] Kattelmann R., Dozier J. (1999). Observations of snowpack ripening in the sierra Nevada, California, u.s.a. J. Glaciology.

[bib63] Kattlemann R. (1997). Destructive Water: Water-Caused Natural Disasters, Their Abatement and Control.

[bib64] Kinar N.J., Pomeroy J.W. (2015). Measurement of the physical properties of the snowpack. Rev. Geophys..

[bib65] Kondragunta C.R., Shrestha K. (2006). Automated real-time operational rain gauge quality-control tools in nws hydrologic operations. Amer. Meteor. Soc,.

[bib66] Ligare S.T., Viers J.H., Null S.E., Rheinheimer D.E., Mount J.F. (2011). Non-uniform changes to whitewater recreation in California’s sierra nevada from regional climate warming. River Res. Appl..

[bib67] Lundquist J.D., Hughes M., Henn B., Gutmann E.D., Livneh B., Dozier J., Neiman P. (2015). High-elevation precipitation patterns: Using snow measurements to assess daily gridded datasets across the sierra nevada, california. J. Hydrometeorology.

[bib68] Lynn E., Cuthbertson A., He M., Vasquez J.P., Anderson M.L., Coombe P., Abatzoglou J.T., Hatchett B.J. (2020). Technical note: Precipitation-phase partitioning at landscape scales to regional scales. Hydrol. Earth Syst. Sci..

[bib69] Marks D., Kimball J., Tingey D., Link T. (1998). The sensitivity of snowmelt processes to climate conditions and forest cover during rain-on-snow: a case study of the 1996 pacific northwest flood. Hydrological Process..

[bib70] Marks D., Link T., Winstral A., Garen D. (2001). Simulating snowmelt processes during rain-on-snow over a semi-arid mountain basin. Ann. Glaciology.

[bib71] Marsh P. (1987). Grain growth in a wet arctic snow cover. Cold Regions Sci. Technology.

[bib72] Marsh P. (1999). Snowcover formation and melt: recent advances and future prospects. Hydrological Process..

[bib73] Marsh P., Woo M.K. (1984). Wetting front advance and freezing of meltwater within a snow cover: 1. observations in the canadian arctic. Water Resour. Res..

[bib74] Mazurkiewicz A.B., Callery D.G., McDonnell J.J. (2008). Assessing the controls of the snow energy balance and water available for runoff in a rain-on-snow environment. J. Hydrol..

[bib75] McCabe G.J., Clark M.P., Hay L.E. (2007). Rain-on-snow events in the western united states. Bull. Am. Meteorol. Soc..

[bib76] McGurk B., Azuma D., Kattelmann R. (1988). Proceedings 56th Western Snow Conference.

[bib77] McGurk B.J. (1986). http://sites/westernsnowconference.org/PDFs/1986McGurk.pdf.

[bib78] Mesinger F., DiMego G., Kalnay E., Mitchell K., Shafran P.C., Ebisuzaki W., Jović D., Woollen J., Rogers E., Berbery E.H. (2006). North american regional reanalysis. Bull. Am. Meteorol. Soc..

[bib79] Musselman K.N., Addor N., Vano J.A., Molotch N.P. (2021). Winter melt trends portend widespread declines in snow water resources. Nat. Clim. Change.

[bib80] Musselman K.N., Clark M.P., Liu C., Ikeda K., Rasmussen R. (2017). Slower snowmelt in a warmer world. Nat. Clim. Change.

[bib81] Musselman K.N., Lehner F., Ikeda K., Clark M.P., Prein A.F., Liu C., Barlage M., Rasmussen R. (2018). Projected increases and shifts in rain-on-snow flood risk over western north america. Nat. Clim. Change.

[bib82] NOAA (2020). NOAA Research and Develoment Vision Areas: 2020-2026. Tech. Rep..

[bib83] Norbiato D., Borga M., Degli Esposti S., Gaume E., Anquetin S. (2008). Flash flood warning based on rainfall thresholds and soil moisture conditions: An assessment for gauged and ungauged basins. J. Hydrol..

[bib84] NRCS (2014). https://directives.sc.egov.usda.gov/viewerFS.aspx?hid=32040.

[bib85] NWS (2020). NWS Building a Weather-Ready Nation: 2019-2022 Strategic Plan. Tech. Rep.

[bib86] O’Hara B.F., Kaplan M.L., Underwood S.J. (2009). Synoptic climatological analyses of extreme snowfalls in the sierra nevada. Weather Forecast..

[bib87] Oakley N.S., Lancaster J.T., Hatchett B.J., Stock J., Ralph F.M., Roj S., Lukashov S. (2018). A 22-year climatology of cool season hourly precipitation thresholds conducive to shallow landslides in california. Earth Interactions.

[bib88] Oakley N.S., Lancaster J.T., Kaplan M.L., Ralph F.M. (2017). Synoptic conditions associated with cool season post-fire debris flows in the transverse ranges of southern california. Nat. Hazards.

[bib89] OECD (2012). https://www.oecd-ilibrary.org/content/publication/9789264122246-en.

[bib90] Osterhuber R. (2009). https://www.sierracollege.edu/ejournals/jscnhm/v2n1/climatesummary.html.

[bib91] Osterhuber R., Schwartz A. (2021). http://datadryad.org/stash/dataset/doi:10.6078/D1941T.

[bib92] Painter T.H., Skiles S.M., Deems J.S., Bryant A.C., Landry C.C. (2012). Dust radiative forcing in snow of the upper colorado river basin: 1. a 6 year record of energy balance, radiation, and dust concentrations. Water Resour. Res..

[bib93] Pedregosa F., Varoquaux G., Gramfort A., Michel V., Thirion B., Grisel O., Blondel M., Prettenhofer P., Weiss R., Dubourg V. (2011). Scikit-learn: Machine learning in Python. J. Machine Learn. Res..

[bib94] Peterson N.R., Brown A.J. (1975). Accuracy of snow measurements, Western Snow Conference, Coronado, California. http://sites/westernsnowconference.org/PDFs/1975Peterson.pdf.

[bib95] Prince H.D., Gibson P.B., DeFlorio M.J., Corringham T.W., Cobb A., Guan B., Ralph F.M., Waliser D.E. (2021). Genesis locations of the costliest atmospheric rivers impacting the western united states. Geophys. Res. Lett.

[bib96] Ralph F.F., Dettinger M., White A., Reynolds D., Cayan D., Schneider T., Cifelli R., Redmond K., Anderson M., Gherke F. (2014). A vision for future observations for western u.s. extreme precipitation and flooding. J. Contemp. Water Res. Education.

[bib97] Ralph F.M., Coleman T., Neiman P.J., Zamora R.J., Dettinger M.D. (2013). Observed impacts of duration and seasonality of atmospheric-river landfalls on soil moisture and runoff in coastal northern california. J. Hydrometeorology.

[bib98] Ralph F.M., Rutz J.J., Cordeira J.M., Dettinger M., Anderson M., Reynolds D., Schick L.J., Smallcomb C. (2019). A scale to characterize the strength and impacts of atmospheric rivers. Bull. Am. Meteorol. Soc..

[bib99] Rössler O., Froidevaux P., Börst U., Rickli R., Martius O., Weingartner R. (2014). Retrospective analysis of a nonforecasted rain-on-snow flood in the alps-a matter of model limitations or unpredictable nature?. Hydrol. Earth Syst. Sci..

[bib100] Schneebeli M. (1995). Development and stability of preferential flow paths in a layered snowpack. IAHS Publications-series Proc. Reports-Intern Assoc Hydrological Sci..

[bib101] Serreze M.C., Clark M.P., Armstrong R.L., McGinnis D.A., Pulwarty R.S. (1999). Characteristics of the western united states snowpack from snowpack telemetry (snotel) data. Water Resour. Res..

[bib102] Siirila-Woodburn E., Rhoades A.M., Hatchett B.J., Huning L., Szinai J., Tague C., Nico P.S., Feldman D., Jones A.D., Collins W.D., Kaatz L. (2021). A low-to-no snow future and its impacts on water resources in the western united states. Nat. Rev. Earth Environ..

[bib103] Singh P., Spitzbart G., Hübl H., Weinmeister H.W. (1997). Hydrological response of snowpack under rain-on-snow events: A field study. J. Hydrol..

[bib104] Song Y., Broxton P.D., Ehsani M.R., Behrangi A. (2021). Assessment of snowfall accumulation from satellite and reanalysis products using SNOTEL observations in alaska. Remote Sensing.

[bib105] Statham G., Haegeli P., Birkeland K.W., Greene E., Israelson C., Tremper B., Stethem C., McMahon B., White B., Kelly J. (2010). International Snow Science Workshop.

[bib106] Steinschneider S., Brown C. (2012). Dynamic reservoir management with real-option risk hedging as a robust adaptation to nonstationary climate. Water Resour. Res..

[bib107] Sterle K., Hatchett B.J., Singletary L., Pohll G. (2019). Hydroclimate variability in snow-fed river systems: Local water managers’ perspectives on adapting to the new normal. Bull. Am. Meteorol. Soc..

[bib108] Sumargo E., Wilson A.M., Ralph F.M., Weihs R., White A., Jasperse J., Asgari-Lamjiri M., Turnbull S., Downer C., Monache L.D. (2020). The hydrometeorological observation network in california’s russian river watershed: Development, characteristics, and key findings from 1997 to 2019. Bull. Am. Meteorol. Soc..

[bib109] Sutcliffe K. (2014). Soil moisture dynamics during snowmelt. https://westernsnowconference.org/sites/westernsnowconference.org/PDFs/2014Sutcliffe.pdf.

[bib110] Sutcliffe K., Clayton J. (2021). Editing soil moisture and soil temperature data at SNOTEL and SCAN sites. Tech. Rep..

[bib111] Uccellini L.W., Ten Hoeve J.E. (2019). Evolving the national weather service to build a weather-ready nation. Bull. Am. Meteorol. Soc..

[bib112] Underwood S.J., Kaplan M.L., King K.C. (2009). The role of upstream midtropospheric circulations in the sierra nevada enabling leeside (spillover) precipitation. part i: A synoptic-scale analysis of spillover precipitation and flooding in a leeside basin. J. Hydrometeorology.

[bib113] Verdhen A. (2018). Rain and snowmelt augmented design flood for highways bridges in snowy mountains. J. Hydrogeological Hydrological Eng..

[bib114] Vicuna S., Leonardson R., Hanemann M.W., Dale L.L., Dracup J.A. (2008). Climate change impacts on high elevation hydropower generation in california’s sierra nevada: a case study in the upper american river. Climatic Change.

[bib115] Wever N., Fierz C., Mitterer C., Hirashima H., Lehning M. (2014). Solving richards equation for snow improves snowpack meltwater runoff estimations in detailed multi-layer snowpack model. The Cryosphere.

[bib116] Wever N., Jonas T., Fierz C., Lehning M. (2014). Model simulations of the modulating effect of the snow cover in a rain-on-snow event. Hydrol. Earth Syst. Sci..

[bib117] Whitaker A.C., Sugiyama H. (2005). Seasonal snowpack dynamics and runoff in a cool temperate forest: lysimeter experiment in niigata, japan. Hydrological Process..

[bib118] White A.B., Anderson M.L., Dettinger M.D., Ralph F.M., Hinojosa A., Cayan D.R., Hartman R.K., Reynolds D.W., Johnson L.E., Schneider T.L. (2013). A twenty-first-century california observing network for monitoring extreme weather events. J. Atmos. Oceanic Technology.

[bib119] White A.B., Moore B.J., Gottas D.J., Neiman P.J. (2019). Winter storm conditions leading to excessive runoff above california’s oroville dam during january and february 2017. Bull. Am. Meteorol. Soc..

[bib120] WMO (2021).

[bib121] Work R.A., Stockwell H.J., Freeman T.G., Beaumont R.T. (1965). https://erdc-library.erdc.dren.mil/jspui/handle/11681/5580.

[bib122] Yan H., Sun N., Wigmosta M., Skaggs R., Hou Z., Leung R. (2018). Next-generation intensity-duration-frequency curves for hydrologic design in snow-dominated environments. Water Resour. Res..

[bib123] Yuba Feather, Steering Committee (2021). https://cw3e.ucsd.edu/FIRO_docs/YF_workplan.pdf.

