# Supplementary material for: Toward snowpack runoff decision support
Source: iScience. 2022 Apr 12;25(5):104240. doi: 10.1016/j.isci.2022.104240 (PMC9051623; doi:10.1016/j.isci.2022.104240)
Supplement: Document S1. Figures S1–S5 [file mmc1.pdf]

iScience, Volume *25*

Supplemental information

Toward snowpack runoff decision support

Anne Heggli, Benjamin Hatchett, Andrew Schwartz, Tim Bardsley, and Emily Hand

Supplemental Information

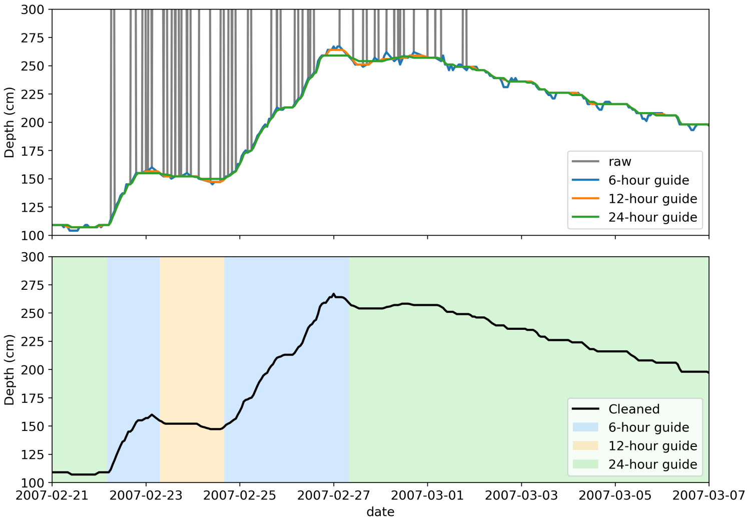

Figure 1: An example of semi-automated cleaning process and results. Raw data represented with grey shows the full-scale readings that occur during snowfall events. The 6-hr guide (blue) removes full-scale readings but experiences diurnal flutter, the 12-hr guide (orange) and the 24-hr guide help reduce diurnal flutter or other sensor noise. Related to STAR Methods

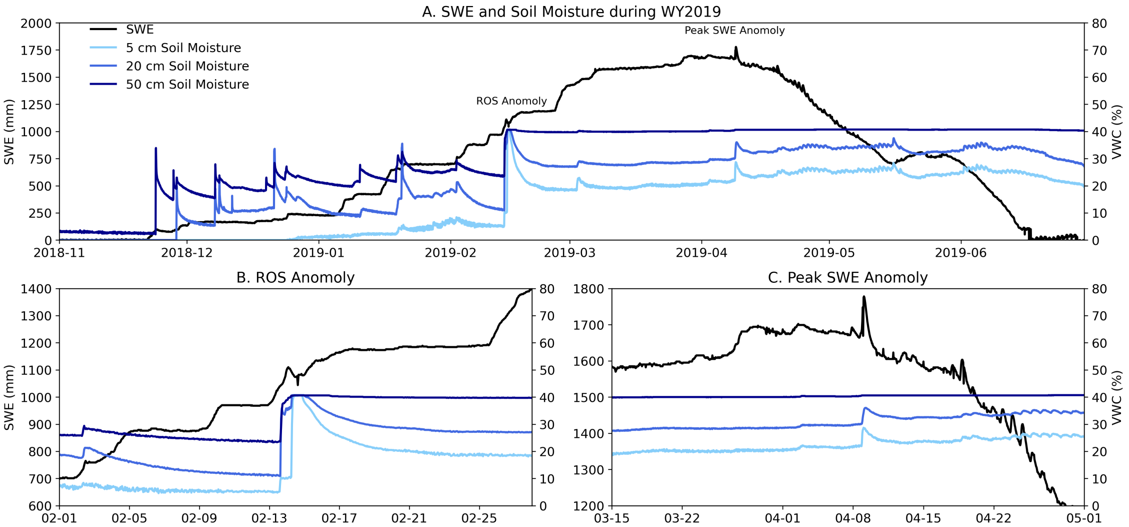

Figure 2: An example of SWE data anomalies and the value of soil moisture data for intersensor comparison. (A) SWE data (black) compared with soil moisture at 5 cm (blue), 20 cm (orange), and 50 cm (green). (B) Rapid increase and decrease of SWE during rainfall event is validated by a rapid increase in soil moisture. (B) A jump in SWE observations corresponds with increase of soil moisture. Related to STAR Methods

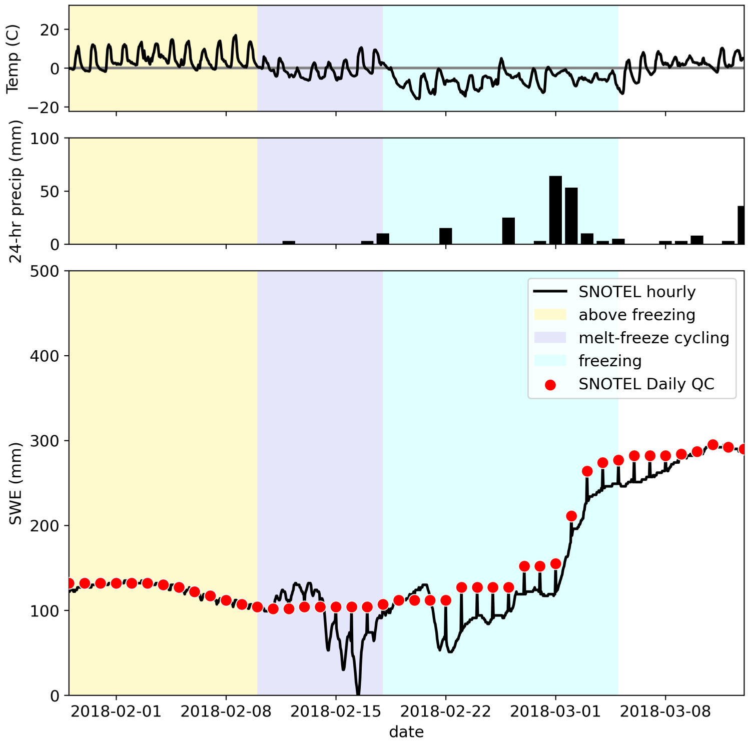

Figure 3: An example of bridging over a snow pillow. An extended period of above-freezing days (yellow) followed by melt-freeze cycles (purple) and sub-freezing temperatures (blue) can cause the formation of complex crust layers that inhibit the transfer of weight to the pillow. The erratic readings from the pillow being during the melt-freeze cycle and persist through the sub-freezing which. There is no significant precipitation to justify the change in SWE and therefore this data should be classified as poor quality. Related to STAR Methods

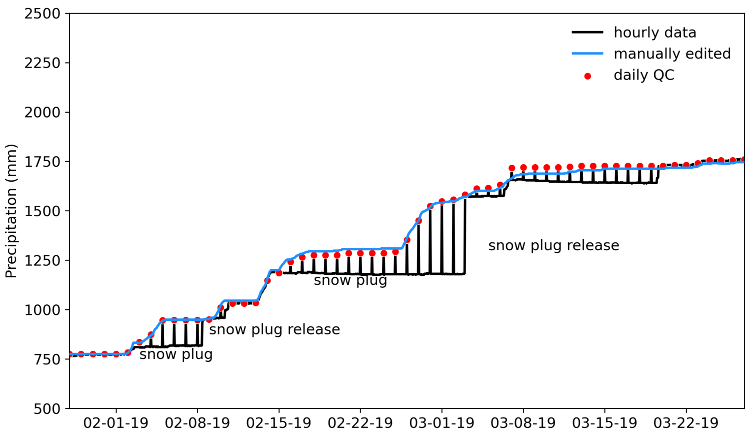

Figure 4: Example of cleaned precipitation data product after snow plug formation. SNOTEL hourly data (black), reviewed SNOTEL daily data (red), cleaned hourly data using the methods outlined in the paper (blue). Related to STAR Methods

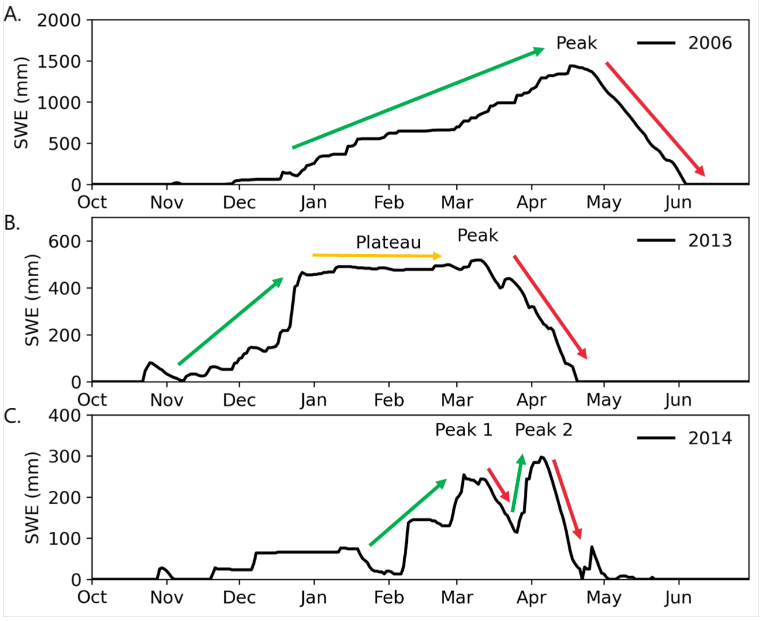

Figure 5: Example of the types of SWE accumulation patterns. Possible SWE accumulation (green) and ablation (red) scenarios. (A) steady accumulation leading to a defined peak prior to ablation period, (B) accumulation with prominent plateau (yellow) prior to ablation, and (C) two peaks with two ablation period melts. Related to STAR Methods
